# On distance-based topological indices and co-indices of fractal-type molecular graphs and their respective graph entropies

**DOI:** 10.1371/journal.pone.0290047

**Published:** 2023-11-09

**Authors:** Mehar Ali Malik, Muhammad Imran, Muhammad Adeel

**Affiliations:** 1 Department of Basic Sciences and Humanities, College of Electrical and Mechanical Engineering, National University of Sciences and Technology (NUST), Rawalpindi, Pakistan; 2 Department of Mathematics, Riphah International University, Lahore, Pakistan; 3 Department of Mathematical Sciences, United Arab Emirates University, Al Ain, United Arab Emirates; University of Naples Federico II, ITALY

## Abstract

In graph theory, a topological index is a numerical value that is in good correlation with certain physical properties of a molecule. It serves as an indicator of how a chemical structure behaves. The Shannon’s entropy describes a comparable loss of data in information transmission networks. It has found use in the field of information theory. Inspired by the concept of Shannon’s entropy, we have calculated some topological descriptors for fractal and Cayley-type dendrimer trees. We also find the entropy that is predicted by these indices.

## 1. Introduction

Graph theory has enabled chemists and other scientists to study the topological characteristics of molecules with the help of graphs. Graph theoretic techniques are used to model molecules and other structures into graphs. A range of topological descriptors are then computed from these molecular graphs to get insights of the structural features of molecules and correlate them with their physical and chemical properties. These topological descriptors offer a better means of comprehending and forecasting the characteristics and bio-activities of these substances.

Modern polymeric substances including dendrimers are man-made, mono-disperse structures which are three dimensional and have branches that resemble trees [1]. Cascade and Arborols are among the others names for dendrimers. Dendrimers come in a variety of forms including chiral, tacto, hybride, peptide, and frechet type dendrimers as well as radially stacked polydendrimers and liquid crytalline [2]. Tomalia introduced the word “dendrimer” to the public for the first time in 1985. It comes from the words “dendrimer” which means “tree” and “meros(part)” [3]. To prevent the synthesis of poly(lysine) dendrimers until the ninth century Denkewalter employed a different strategy [4]. Fritz Vögtle [5] created the first dendrimers in 1978 using divergent synthesis techniques.

Topological indices are crucial in modeling various characteristics and activities of molecules without the use of a weight lab in quantitative structure-property/activity relationship (QSPR/QSAR) studies [6]. The benzenoid hydrocarbon molecular structure is shown by caterpillar trees. In 2013, researchers analyzed and calculated the first and second Zagreb indices of star-like tree graphs, sun-like graphs and caterpillar trees that included hydrocarbons, particularly ethane, propane and butane [7].

## 2. Study of topological indices and their applications

In Mathematical chemistry, molecular topology and chemical graph theory, a topological index is a function that gives us a numerical value associated with a molecule’s chemical structure. Chemical graph theory describes how that particular molecular structure came to be. Topological index provides the most straightforward way to translate the chemical structure into a numerical number. Applications of distance-based topological indices are described as they link many physical and chemical parameters such as boiling point, melting point, resonance energy, strain energy and so forth.

A topological index is a type of a molecular descriptor that is calculated based on the molecular graph of a chemical compound [8]. Topological indices are numerical parameters of a graph which characterize its topology. Topological indices are used, for example, in the development of quantitative structure-activity relationships (QSAR’s) in which biological activities or other properties of molecules are correlated with their chemical structure [9].

Hosoya index was the first topological index recognized in chemical graph theory [10]. Wiener index (also called Wiener number) was introduced by Harold Wiener is a topological index defined as the sum of lengths of the shortest paths between all pairs of vertices in the chemical graph representing the non-hydrogen atoms in a molecule [11]. Wiener introduced it in 1947 and at that time Wiener called it “path number” [12].

$$\begin{array}{c} W\left(G\right)=\frac{1}{2}\sum_{v\in V(G)}d_{G}\left(v\right), \end{array}$$ (1)

where *d*_*G*_(*v*) denotes the sum of all distances starting from *v* in *G*. The first and second Zagreb indices were introduced more than 50 years back by Gutman and Trinajestić in 1972 [13]. First and Second Zagreb Indices are found to be helpful for calculation of the total *π*-electron energy of molecular orbitals [14]. First Zagreb index is defied as the sum of squares of vertex degrees in *G*.

$$\begin{array}{c} M_{1}\left(G\right)=\sum_{v\in V(G)}d^{2}\left(v\right). \end{array}$$ (2)

The second Zagreb index is defined as follows.

$$\begin{array}{c} M_{2}\left(G\right)=\sum_{vu\in V(G)}d\left(v\right)d\left(u\right). \end{array}$$ (3)

The Zagreb indices were reformulated as Zagreb co-indices by Ashrafi et al. [15] in 2010 as follows.

$$\begin{array}{ccc} \bar{M_{1}}\left(G\right) & = & \sum_{uv\notin E(G)}\left[d\left(u\right)+d\left(v\right)\right] \end{array}$$ (4)

$$\begin{array}{ccc} \bar{M_{2}}\left(G\right) & = & \sum_{uv\notin E(G)}d\left(u\right)d\left(v\right). \end{array}$$ (5)

Recently introduced leap Zagreb indices of a graph based on the second degrees of vertices (number of their second neighbors). The first leap Zagreb index *LM*_1_(*G*) is equal to the sum of squares of the second degrees of the vertices, the second leap Zagreb index *LM*_2_(*G*) is equal to the sum of the products of the second degrees of pairs of adjacent vertices of G and the third leap Zagreb index *LM*_3_(*G*) is equal to the sum of the products of the first degrees with the second degrees of the vertices [16]. Simultaneously leap first, second and third Zagreb indices are defined below.

$$\begin{array}{c} LM_{1}=\sum_{u\in V(G)}d_{2}^{2}\left(u\right) \end{array}$$ (6)

$$\begin{array}{c} LM_{2}=\sum_{uv\in E(G)}d_{2}\left(u\right)d_{2}\left(v\right) \end{array}$$ (7)

$$\begin{array}{c} LM_{3}=\sum_{u\in V(G)}d_{1}\left(u\right)d_{2}\left(u\right), \end{array}$$ (8)

where *d*_2_(*v*) denotes the number of vertices of *G* that are at distance 2 from *v*. In 2018, Kulli et al. [17] defined first and second leap hyper-Zagreb indices. First and Second leap hyper-Zagreb indices are defined as follows.

$$\begin{array}{ccc} HLM_{1}\left(G\right) & = & \sum_{uv\in E(G)}{\left[d_{2}\left(u\right)+d_{2}\left(v\right)\right]}^{2} \end{array}$$ (9)

$$\begin{array}{ccc} HLM_{2}\left(G\right) & = & \sum_{uv\in E(G)}{\left[d_{2}\left(u\right)d_{2}\left(v\right)\right]}^{2}. \end{array}$$ (10)

The concept of co-indices was first discussed by Doslić in 2008, and was first applied to the two Zagreb indices [18]. The second ($\bar{LM_{2}}$) and third ($\bar{LM_{3}}$) versions of the leap Zagreb co-indices are defined for any graph as follows.

$$\begin{array}{c} \bar{LM_{2}}\left(G\right)=\sum_{uv\notin E(G)}d_{2}\left(u\right)d_{2}\left(v\right) \end{array}$$ (11)

$$\begin{array}{c} \bar{LM_{3}}\left(G\right)=\sum_{uv\notin E(G)}\left(d_{2}\left(u\right)+d_{2}\left(v\right)\right). \end{array}$$ (12)

Futula and Gutman studied a vertex degree-based topological index called forgotten index in 2015 [19] which was introduced more than 40 years ago by Gutman and Trinajstić in 1972. It is defined as

$$\begin{array}{c} F\left(G\right)=\sum_{uv\in E(G)}\left(d^{2}\left(u\right)+d^{2}\left(v\right)\right) \end{array}$$ (13)

another form of this index is

$$\begin{array}{c} F\left(G\right)=\sum_{v\in V(G)}d^{3}\left(v\right). \end{array}$$ (14)

Forgotten co-index is another useful topological descriptor among other co-indices but it is less explored as compared to other co-indices. Forgotten co-index has found applications in material engineering, pharmaceuticals and in general chemical industry [20]. It is defined as

$$\begin{array}{c} \bar{F}\left(G\right)=\sum_{uv\notin E(G)}\left[d^{2}\left(u\right)+d^{2}\left(v\right)\right]. \end{array}$$ (15)

Wiener polarity index, Lukovits and Linert demonstrated quantitative structure property relationships in a series of a-cyclic and cycle-containing hydrocarbons [21]. Recently, Du et al. [22] described a linear time algorithm for computing the Wiener polarity index of trees and characterized the trees maximizing the index among all trees of a given order. Deng [23] also gave the extremal Wiener polarity index of all chemical trees with order *n*. The Wiener polarity index *W*_*p*_(*G*) of an *n*-vertex molecular graph is the number of unordered pairs of vertices *u*, *v* in *G* where the distance between *u* and *v* is 3. The Wiener polarity index is defined for any graph as follows.

$$\begin{array}{c} W_{p}\left(G\right)=\frac{1}{2}\sum_{v\in V(G)}d_{3}\left(v\right), \end{array}$$ (16)

where *d*_3_(*v*) denotes the number of vertices of *G* that are at distance 3 from *v*.

In 2005, researchers looked at the topological indices of well-known dendrimers [24]. In 2017, authors computed topological indices of the line graphs of the Banana tree graphs and the firecracker graphs [25]. Azeem et al. [26–30] studied some metric type topological invariants of several graph families. Dobrynin et al. [31, 32] studied the Wiener index of hexagonal chains. Kamran et al. [33, 34] studied topological indices such as Wiener index, terminal Wiener index of chemical graphs.

## 3. Motivation

This article included with the discussion about degree based topological indices and also some articles and paper work of different authors. We will share some of observations about degree based topological indices and there importance in different fields. However we know in past many vertex degree-based graph invariants also known as vertex-degree-based topological indices have been presented and thoroughly investigated in the mathematical and chemical literature.

Zagreb connection index is determined by the connection number (degree of vertices at distance 2) for the resulting graphs generated by the corona cartesian and lexicographic product operations. Only a few instances of how the observations are applied to particular chemical structures include alkanes cycloalkanes linear polynomial chains carbon nanotubes fences and closed fences [35]. M-polynomial a special algebraic polynomial is used to determine the expressions of several degree-based topological indices [36]. Zagreb index is the most used index for assessing physical and chemical properties of molecules [37].

It’s interesting to note that the first leap Zagreb index strongly correlates with the bolling point, entropy, DHVAP, HVAP, and eccentric factor of chemical compounds [38]. First and second Zagreb indices and co-indices of a graph and its complements are constructed, together with a full set of relations between them. Several derived graph’s first Zagreb index formulas are also obtained. First Zagreb co-indices of a graph and its complement are always equal is an impressive outcome [39].

Divergent growth and convergent growth techniques are used to synthesise dendrimers. The divergent method indicates that the synthesis of a dendrimer proceeds step by step starting with an activated core, *B*_*n*_ (*n* ≥ 2) to which the subsequent dendrimer ages are created by the sequential attachment of components known as monomers (pattern 1). The monomers used are some variety of *AB*_*n*_ (*n* ≥ 2), where *A* and *B*_*n*_ refer to two different types of beneficial groups. The *B*_*n*_ useful gathering of the *AB*_*n*_ monomer is disabled/secured, allowing for the constrained growth of the dendrimer, in contrast to the responsive *A*-useful gathering of the *AB*_*n*_ monomer.

Through a synthetic bond arrangement between the monomer’s *A*-utilitarian gathering and one of the substrate’s activated *B*-utilitarian gathers, the monomers are connected to a substrate particle (a growing dendrimer). Their activation could well be directed by the evacuation of assuring groups or the coupling of the *B*-functionalities with a subsequent particle. The most often employed monomers are *AB*_2_ tri-functional monomers.

**Fig 1:**
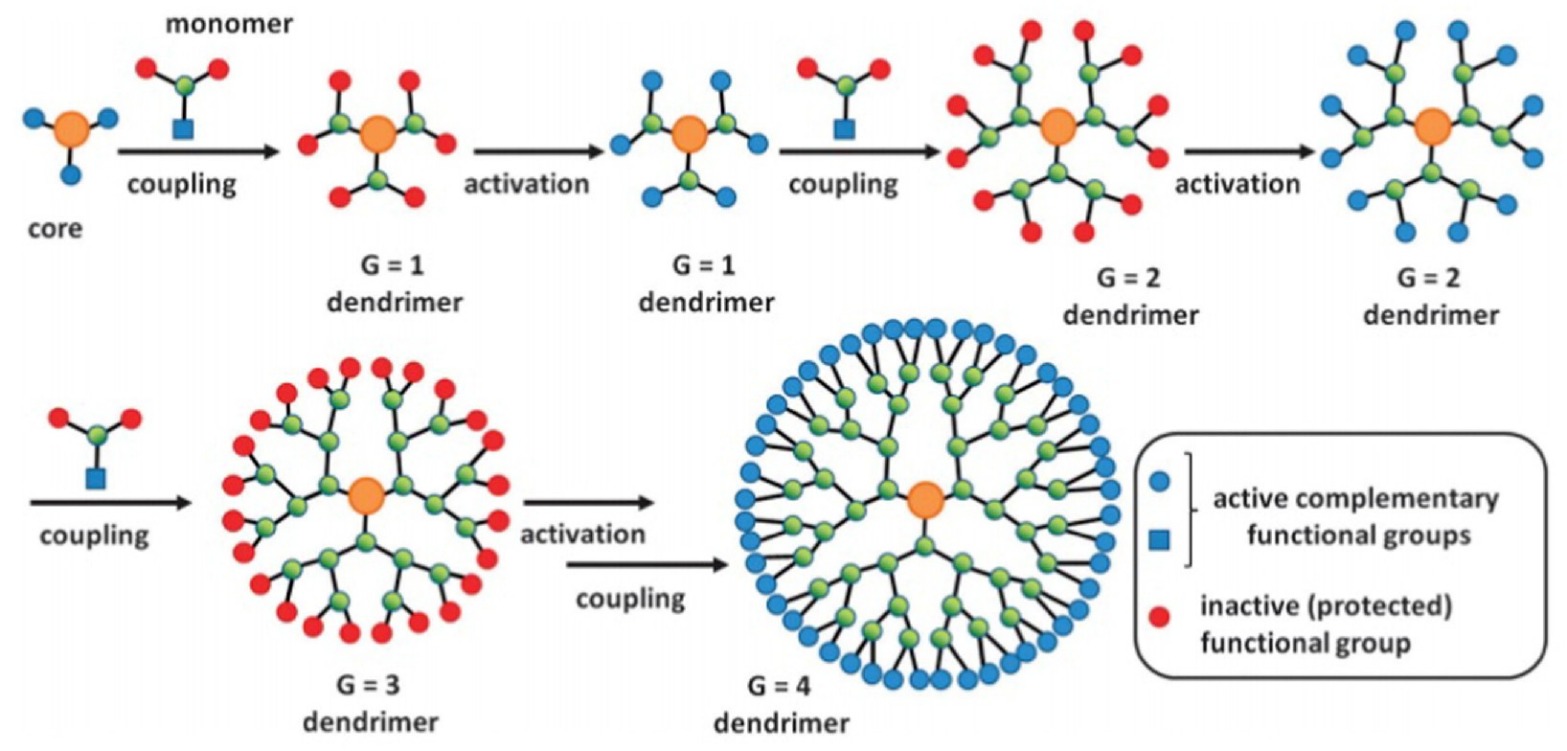
The divergent growth method.

A distinct dendrimer is created once these monomers connect to the centre. A second age dendrimer is produced by the two subsequent steps, which are reliant on the activation of the *B* functions on the first dendrimer and their coupling with a different configuration of monomers. The redundancy of these two stages allows for the attainment of the required increased age dendrimer [40], as seen in Fig 1.

**Fig 2:**
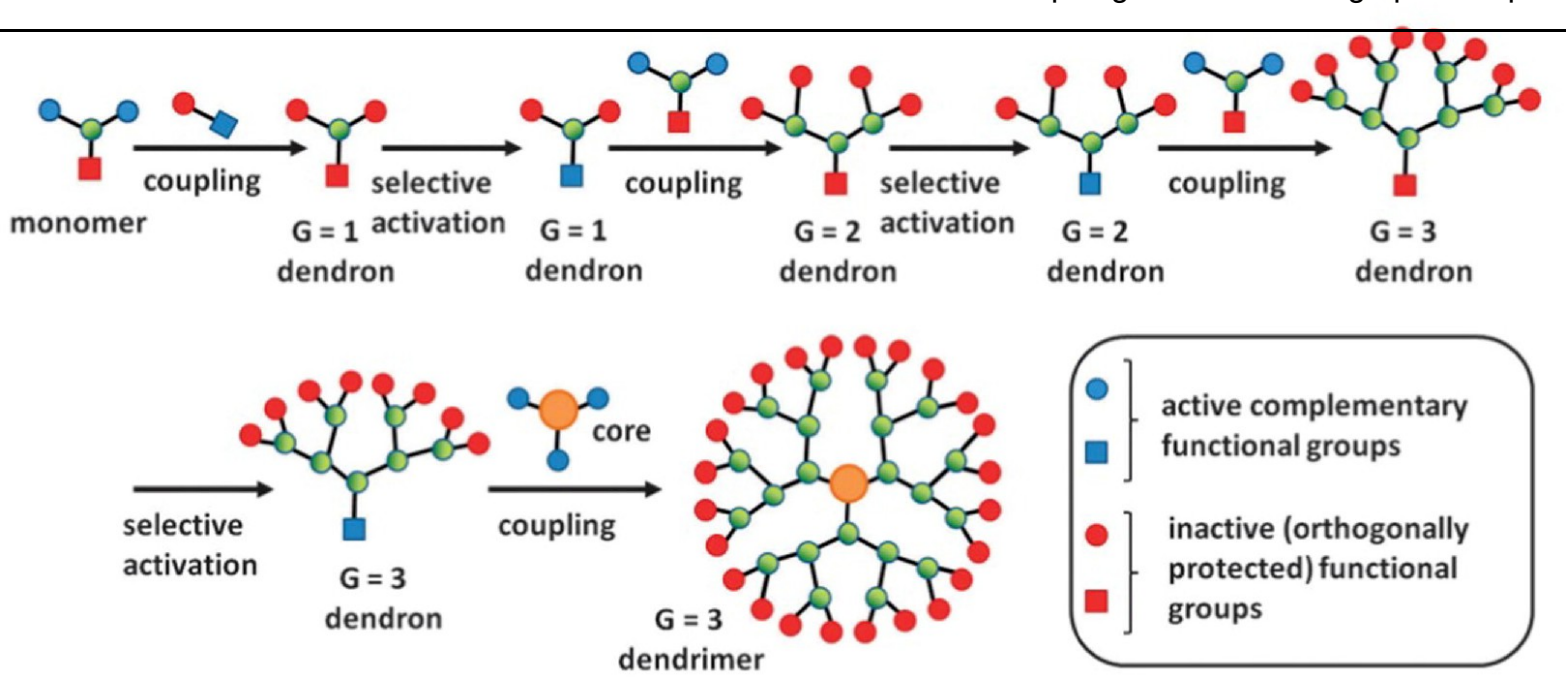
The convergent growth method.

The convergent strategy was initially introduced by Hawker and Frechet in 1989 − 1990, and it is an elective course for creating dendrimers. Dendrons, specific dendrimer wedges, are first orchestrated and then coupled to an active core (pattern 2). Dendron combination is controlled by the regular *AB*_2_ monomer, which possesses sensitive B-functionalities and disabled/ensured A-usefulness (see Fig 2).

In the first step, the monomer is treated to a reaction with a molecule that would eventually create the edges of the dendrimer (outside). Often, the biggest development is the presentation of secure bunches on monomer B-functionalities. Consequently, it is feasible to create a unique dendron (*G*_1_). The last two steps, which lead to the formation of a (*G*_2_) dendron, are the activation of a dendron point of convergence and its coupling with the *AB*_2_ monomers. The dendron age increases with each repetition of these two procedures, as seen in the accompanying graph [40].

The idea of entropy was initially presented by Shannon in 1948. It measures the unpredictability of an information system’s content. The use of this concept in chemical networks and graphs came later. Entropy of a graph was established by Rashevsky [41]. Probability distributions are correlated with graph invariants (vertices, edges, etc.) via intrinsic and extrinsic graph entropy measurements (Mowshowitz and Dehmer 2012, [42]). The information entropy studied by Chen et al. in 2014 [43] based on Shannon’s entropy (Shannon 1948, [44]) is described as:

$$\begin{array}{ccc} E_{w}\left(Z\right) & = & -\sum_{j=1}^{m}F_{j}\frac{w(x_{j}y_{j})}{T_{d}}\;{\text{log}}_{2}\;\frac{w(x_{j}y_{j})}{T_{d}} \end{array}$$ (17)

$$\begin{array}{ccc} & = & \text{log}\left(T_{d}\right)-\frac{1}{T_{d}}\sum_{j=1}^{m}F_{j}w(x_{j}y_{j}\;{\text{log}}_{2}\;w\left(x_{j}y_{j}\right), \end{array}$$ (18)

where $T_{d}=\sum_{j=1}^{m}$ *F*_*j*_*w*(*x*_*j*_*y*_*j*_) tells us about the topological descriptor, *w*(*xy*) is the weight of the edge, *m* is the number of edge types and *F*_*j*_ represents the frequency or number of repeats. Recently in 2022, Imran et al. [45] studied entropy measures of some molecular graphs of nano dendrimers.

## 4. Graphical structure of fractal trees *F*_*r*_

The Latin root of the term “fractal” means “to break.” Fractals are geometric patterns in which every smaller component shares characteristics with the entire structure. Fractals are graphical structures that consist of smaller curves or patterns with precisely the same form. An approximation of a fractal pattern may be seen in how a tree trunk divides into shorter and shorter branches and limbs. The Romanesco broccoli, lightning bolt, angelica flower-head, and Von Koch snowflake are just a few more fractal examples. Numerous objects, such as the Sierpinski triangle, the von koch curve, broccoli, ferns, and the white lotus flower, are instances of fractals.Iterative construction is used to create fractal tree *F*_*r*_.

**Fig 3:**
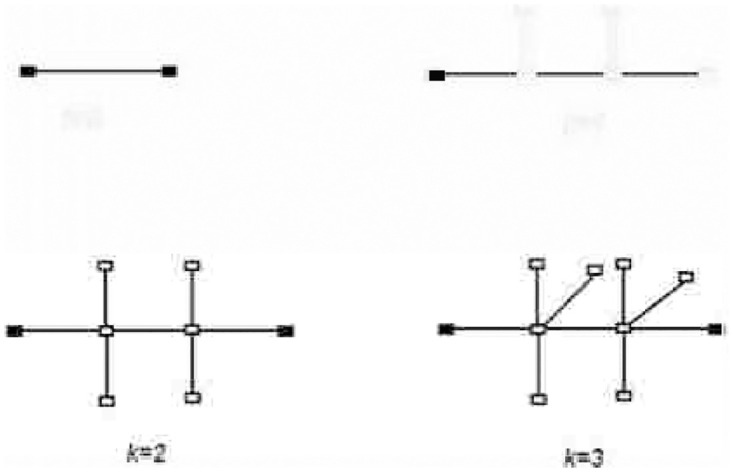
Model for expansion of *F*_*r*_.

**Fig 4:**
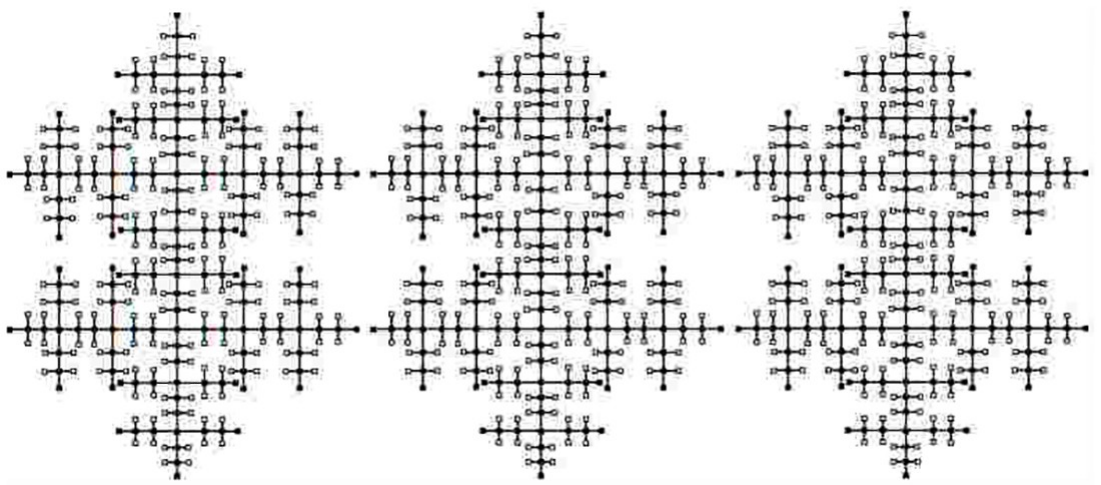
The representation of *F*_3_ for *k* = 2.

When *r* ⩾ 1 represents the iteration number starting with *F*_1_. In the very first there is only one edge between any two nodes. We create *F*_*r*_ for different values of *k* as shown in the Fig 3. First we put two new nodes on each edge to establish a path of length three. Next step is to add *K* additional vertices, one for each of the two middle vertices and then join them to the central vertices. Fig 4 accordingly shows the corresponding fractal trees *F*_3_, for *k* = 2.

## 5. Main results

Now we present our main results related to degree and distance-based topological indices of fractal and Cayley-type trees.

## 6. First, second and third degrees of vertices in *F*_*r*_

In this section, we are looking forward for first, second and third degrees of vertices of *F*_*r*_. The order and size of the dendrimers *F*_*r*_ is given in Table 1. The first, second and third degrees for vertices are given in Tables 2–4. The edge partitions with respect to first degrees (*d*(*u*), *d*(*v*)), second degrees (*d*_2_(*u*), *d*_2_(*v*)) and first-second degrees (*d*(*u*), *d*_2_(*v*)) of end-vertices of edges are given in Tables 5–7. The degree partition of vertex set of the complement of *F*_*r*_ is given in Table 8.

**Table 1:** The order and size of *F*_*r*_.

Order of *F*_*r*_	Size of *F*_*r*_
7^*r*^(2*k* + 3) + 1	7^*r*^(2*k* + 3)

**Table 2:** The first degree vertex partition of *F*_*r*_.

*d*(*v*)	Frequency
1	$\frac{1}{3}\left[7^{r}\left(3k+1\right)2+4\right]$
4	$\frac{1}{3}\left(7^{r}-1\right)$
*k* + 2	2(7^*r*^)

**Table 3:** The second degree vertex partition of *F*_*r*_.

*d*_2_(*v*)	Frequency
*k* + 1	$\frac{8}{3}\left[7^{r-1}\left(6k+2\right)+1\right]$
*k* + 4	$\frac{4}{3}\left[7^{r}-1\right]$
4*k* + 4	$\frac{1}{3}\left[7^{r}-1\right]$

**Table 4:** The 3^*rd*^ degree vertex partition of *F*_*r*_.

*d*_3_(*v*)	Frequency
3	$\frac{1}{3}\left[7^{r-1}\left(3k+1\right)2+4\right]$
*k* + 1	[7^*r*−1^(3 *k* + 1)2 + 4]
*k* + 4	$\frac{8}{3}\left[7^{r}-1\right]$
3*k* + 3	$\frac{1}{3}\left[7^{r-1}\left(3k+1\right)2+4\right]$
3*k* + 6	$\frac{1}{3}\left[7^{r-1}\left(3k+1\right)2-8\right]$
4*k* + 4	$\frac{1}{3}\left[7^{r-1}\left(3k+1\right)-1\right]$

### 6.1 Wiener-type indices of *F*_*r*_

In this section we will investigate the Wiener index and Wiener polarity index of graphs *F*_*r*_.

**Theorem 6.1**. *Wiener index of Graph F*_*r*_ *is denoted by W*(*F*_*r*_) *and is given as*.

$$\begin{array}{c} W\left(F_{r}\right)=7^{r}\left(2k+3\right). \end{array}$$

*Proof*. We use Table 2 as follows.

$$\begin{array}{ccc} W(F_{r}) & = & \frac{1}{2}\sum_{v\in V(F_{r})}d_{F_{r}}\left(v_{j}\right)\\ & = & \frac{1}{2}[\left(\frac{1}{3}\right)\left(7^{r}\left(3k+1\right)2+4\right)+\left(4\right)\left(\frac{1}{3}\right)\left(7^{r}-1\right)\\ & & +\left(k+2\right)\left(2\right)\left(7^{r}\right)]\\ W(F_{r}) & = & 7^{r}\left(2k+3\right). \end{array}$$

The Eq (17) and Table 1 gives

$$\begin{array}{ccc} E_{W}\left(F_{r}\right) & = & \text{log}[7^{r}\left(2k+3\right)]\\ & & -\frac{\frac{1}{3}\left[7^{r}\left(3k+1\right)2+4\right]\left(1.5\right)\text{log}\left(1.5\right)}{7^{r}\left(2k+3\right)}\\ & & -\frac{\frac{1}{3}\left[7^{r}-1\right]\left(2\right)\text{log}\left(2\right)}{7^{r}\left(2k+3\right)}\\ & & -\frac{2\left[7^{r}\right]\left(\frac{k+2}{2}\right)\text{log}\left(\frac{k+2}{2}\right)}{7^{r}\left(2k+3\right)}, \end{array}$$

which completes the proof.

In the next theorem, we will discuss Wiener polarity index of *F*_*r*_ with the help of table and basic results.

**Theorem 6.2**. *Wiener Polarity index of Graph F*_*r*_ *is denoted by W*_*p*_(*F*_*r*_) *and is given as*.

$$\begin{array}{c} W_{p}\left(F_{r}\right)=7^{r-1}\left(11k^{2}+30k+43\right)-2\left(k+4\right). \end{array}$$

*Proof*. We use Table 4 as follows.

$$\begin{array}{ccc} W_{p}\left(F_{r}\right) & = & \frac{1}{2}\sum_{j=1}^{n}d_{3}\left(v_{j}\left(F_{r}\right)\right)\\ & = & \frac{1}{2}[3\left(\frac{1}{3}\right)\left(7^{r-1}\left(3k+1\right)2+4\right)+\left(k+1\right)\left(7^{r-1}\left(3k+1\right)2+4\right)\\ & & +\left(k+4\right)\frac{8}{3}\left(7^{r}-1\right)+\left(3k+3\right)\frac{1}{3}\left(7^{r-1}\left(3k+1\right)2+4\right)\\ & & +\left(3k+6\right)\frac{1}{3}\left(7^{r-1}\left(3k+1\right)2-8\right)+\left(4k+4\right)\frac{1}{3}\left(7^{r-1}\left(3k+1\right)-1\right)]\\ W_{p}\left(F_{r}\right) & = & 7^{r-1}\left(11k^{2}+30k+43\right)-2\left(k+4\right). \end{array}$$

The Eq (17) and Table 4 gives

$$\begin{array}{ccc} E_{W_{p}}\left(F_{r}\right) & = & \text{log}[7^{r-1}\left(11k^{2}+30k+43\right)-2\left(k+4\right)]\\ & & -\frac{\frac{1}{3}\left[7^{r-1}\left(3k+1\right)2+4\right]\left(1.5\right)\text{log}\left(1.5\right)}{7^{r-1}\left(11k^{2}+30k+43\right)-2\left(k+4\right)}\\ & & -\frac{\left[7^{r-1}\left(3k+1\right)2+4\right]\left(\frac{k+1}{2}\right)\text{log}\left(\frac{k+1}{2}\right)}{7^{r-1}\left(11k^{2}+30k+43\right)-2\left(k+4\right)}\\ & & -\frac{\frac{8}{3}\left[7^{r}-1\right]\left(\frac{k+4}{2}\right)\text{log}\left(\frac{k+4}{2}\right)}{7^{r-1}\left(11k^{2}+30k+43\right)-2\left(k+4\right)}\\ & & -\frac{\frac{1}{3}\left[7^{r-1}\left(3k+1\right)2+4\right]\left(\frac{3k+3}{2}\right)\text{log}\left(\frac{3k+3}{2}\right)}{7^{r-1}\left(11k^{2}+30k+43\right)-2\left(k+4\right)}\\ & & -\frac{\frac{1}{3}\left[7^{r-1}\left(3k+1\right)2-8\right]\left(\frac{3k+6}{2}\right)\text{log}\left(\frac{3k+3}{2}\right)}{7^{r-1}\left(11k^{2}+30k+43\right)-2\left(k+4\right)}\\ & & -\frac{\frac{1}{3}\left[7^{r-1}\left(3k+1\right)-1\right]\left(2k+2\right)\text{log}\left(2k+2\right)}{7^{r-1}\left(11k^{2}+30k+43\right)-2\left(k+4\right)}, \end{array}$$

and the proof follows.

### 6.2 Zagreb-type indices of *F*_*r*_

In the very first stage we are going to calculate the leap Zagreb indices of first second and third versions for *F*_*r*_. After that we will discuss other indices.

**Theorem 6.3**. *The First Leap Zagreb Index of Fractal tree F*_*r*_ *is denoted by LM*_1_(*F*_*r*_) *and is given as*

$$\begin{array}{c} LM_{1}\left(F_{r}\right)=4\left[7^{r-1}\left(4k^{3}+21k^{2}+44k+48\right)-\left(k^{2}+4k+6\right)\right]. \end{array}$$

*Proof*. Using the values from Table 3, we get

$$\begin{array}{ccc} LM_{1}\left(F_{r}\right) & = & \sum_{j=1}^{n}d_{2}\left(v_{j}\left(F_{r}\right)\right)\\ & = & {\left(k+1\right)}^{2}\frac{8}{3}\left[7^{r-1}\left(3k+1\right)2+1\right]+{\left(k+4\right)}^{2}\frac{4}{3}\left[7^{r}-1\right]\\ & & +{\left(4k+4\right)}^{2}\frac{1}{3}\left[7^{r}-1\right]\\ LM_{1}\left(F_{r}\right) & = & 4[7^{r-1}\left(4k^{3}+21k^{2}+44k+48\right)-\left(k^{2}+4k+6\right)]. \end{array}$$

By Eq (17) and Table 3, we get

$$\begin{array}{ccc} E_{LM_{1}}\left(F_{r}\right) & = & \text{log}[4\left(7^{r-1}\left(4k^{3}+21k^{2}+44k+48\right)-\left(k^{2}+4k+6\right)\right)]\\ & & -\frac{\left[\frac{8}{3}\left(7^{r-1}\left(3k+1\right)2+1\right)\right]\left(k^{2}+2k+1\right)\text{log}\left(k^{2}+2k+1\right)}{4[7^{r-1}\left(4k^{3}+21k^{2}+44k+48\right)-\left(k^{2}+4k+6\right)]}\\ & & -\frac{\left[\frac{4}{3}\left(7^{r}-1\right)\right]\left(k^{2}+8k+16\right)\text{log}\left(k^{2}+8k+16\right)}{4[7^{r-1}\left(4k^{3}+21k^{2}+44k+48\right)-\left(k^{2}+4k+6\right)]}\\ & & -\frac{\left[\frac{1}{3}\left(7^{r}-1\right)\right]\left(16k^{2}+32k+16\right)\text{log}\left(16k^{2}+32k+16\right)}{4[7^{r-1}\left(4k^{3}+21k^{2}+44k+48\right)-\left(k^{2}+4k+6\right)]}. \end{array}$$

**Theorem 6.4**. *The Second Leap Zagreb Index of Fractal tree F*_*r*_ *is denoted by LM*_2_(*F*_*r*_) *and is given as*

$$\begin{array}{c} LM_{2}\left(F_{r}\right)=7^{r-1}\left(6k^{3}+332k+282\right)+7^{r}\left(11k^{2}\right)-4\left(k^{2}+9k+11\right). \end{array}$$

*Proof*. We use Table 6 as follows.

$$\begin{array}{ccc} LM_{2}\left(F_{r}\right) & = & \sum_{j=1}^{n}d_{2}\left(u_{j}\left(F_{r}\right)\right)d_{2}\left(v_{j}\left(F_{r}\right)\right)\\ & = & \left(k+1\right)\left(k+1\right)\left[7^{r-1}\left(3k+1\right)2+4\right]+\left(k+1\right)\left(k+4\right)\frac{1}{3}\left[10\left(7^{r}\right)-4\right]\\ & & +\left(k+4\right)\left(k+4\right)\frac{1}{3}\left[7^{r}-4\right]+\left(k+4\right)\left(4k+4\right)\frac{4}{3}\left[7^{r}-1\right]\\ LM_{2}\left(F_{r}\right) & = & 7^{r-1}\left(6k^{3}+332k+282\right)+7^{r}\left(11k^{2}\right)-4\left(k^{2}+9k+11\right). \end{array}$$

Using Eq (17) and Table 6 gives

$$\begin{array}{ccc} E_{LM_{2}}\left(F_{r}\right) & = & \text{log}[7^{r-1}\left(6k^{3}+332k+282\right)+7^{r}\left(11k^{2}\right)-4\left(k^{2}+9k+11\right)]\\ & & -\frac{\left[7^{r-1}\left(3k+1\right)2+4\right]\left(k^{2}+2k+1\right)\text{log}\left(k^{2}+2k+1\right)}{7^{r-1}\left(6k^{3}+332k+282\right)+7^{r}\left(11k^{2}\right)-4\left(k^{2}+9k+11\right)}\\ & & -\frac{\left[\frac{1}{3}\left(10\left(7^{r}\right)-4\right)\right]\left(k^{2}+5k+4\right)\text{log}\left(k^{2}+5k+4\right)}{7^{r-1}\left(6k^{3}+332k+282\right)+7^{r}\left(11k^{2}\right)-4\left(k^{2}+9k+11\right)}\\ & & -\frac{\left[\frac{1}{3}\left(7^{r}-4\right)\right]\left(k^{2}+8k+16\right)\text{log}\left(k^{2}+8k+16\right)}{7^{r-1}\left(6k^{3}+332k+282\right)+7^{r}\left(11k^{2}\right)-4\left(k^{2}+9k+11\right)}\\ & & -\frac{\left[\frac{4}{3}\left(7^{r}-1\right)\right]\left(4k^{2}+20k+16\right)\text{log}\left(4k^{2}+20k+16\right)}{7^{r-1}\left(6k^{3}+332k+282\right)+7^{r}\left(11k^{2}\right)-4\left(k^{2}+9k+11\right)}. \end{array}$$

**Table 5:** The first degrees of end-vertices of edges in *F*_*r*_.

(*d*(*u*), *d*(*v*))	Frequency
1, *k* + 2	$\frac{1}{3}\left[7^{r}\left(3k+2\right)2+4\right]$
4, *k* + 2	$\frac{4}{3}\left[7^{r}-1\right]$
*k* + 2, *k* + 2	7^r^

**Table 6:** The second degrees of end-vertices of edges in *F*_*r*_.

(*d*_2_(*u*), *d*_2_(*v*))	Frequency
*k* + 1, *k* + 1	[7^*r*−1^(3 *k* + 1)2 + 4]
*k* + 1, *k* + 4	$\frac{1}{3}\left[10\left(7^{r}\right)-4\right]$
*k* + 4, *k* + 4	$\frac{1}{3}\left[7^{r}-4\right]$
*k* + 4, 4*k* + 4	$\frac{4}{3}\left[7^{r}-1\right]$

**Theorem 6.5**. *The Third Leap Zagreb Index of Fractal tree F*_*r*_ *is denoted by LM*_3_(*F*_*r*_) *and is given as*

$$\begin{array}{c} LM_{3}\left(F_{r}\right)=7^{r-1}\left(2k^{3}+46k^{2}+102k+86\right)-8k-12. \end{array}$$

*Proof*. We use Table 7 as follows.

$$\begin{array}{ccc} LM_{3}\left(F_{r}\right) & = & \sum_{j=1}^{n}d_{1}\left(v_{j}\left(F_{r}\right)\right)d_{2}\left(v_{j}\left(F_{r}\right)\right)\\ & = & \left(1\right)\left(k+1\right)\frac{1}{3}\left[7^{r}\left(3k+1\right)2+4\right]+\left(k+2\right)\left(k+1\right)\frac{1}{3}\left[7^{r-1}\left(3k+1\right)2+4\right]\\ & & +\left(k+2\right)\left(k+4\right)\frac{4}{3}\left[7^{r}-1\right]+\left(4\right)\left(4k+4\right)\frac{1}{3}\left[7^{r-1}\left(3k+1\right)-1\right]\\ LM_{3}\left(F_{r}\right) & = & 7^{r-1}\left(2k^{3}+46k^{2}+102k+86\right)-8k-12. \end{array}$$

The Eq (17) and Table 7 gives

$$\begin{array}{ccc} E_{LM_{3}}\left(F_{r}\right) & = & \text{log}[7^{r-1}\left(2k^{3}+46k^{2}+102k+86\right)-8k-12]\\ & & -\frac{[\frac{1}{3}\left(7^{r}\left(3k+1\right)2+4\right)]\left(k+1\right)\text{log}\left(k+1\right)}{7^{r-1}\left(2k^{3}+46k^{2}+102k+86\right)-8k-12}\\ & & -\frac{\left[\frac{1}{3}\left(7^{r-1}\left(3k+1\right)2+4\right)\right]\left(k^{2}+3k+2\right)\text{log}\left(k^{2}+3k+2\right)}{7^{r-1}\left(2k^{3}+46k^{2}+102k+86\right)-8k-12}\\ & & -\frac{\left[\frac{4}{3}\left(7^{r}-1\right)\right]\left(k^{2}+6k+8\right)\text{log}\left(k^{2}+6k+8\right)}{7^{r-1}\left(2k^{3}+46k^{2}+102k+86\right)-8k-12}\\ & & -\frac{[\frac{1}{3}\left(7^{r-1}\left(3k+1\right)-1\right)]\left(16k+16\right)\text{log}\left(16k+16\right)}{7^{r-1}\left(2k^{3}+46k^{2}+102k+86\right)-8k-12}. \end{array}$$

**Table 7:** The first and second degrees of vertices in *F*_*r*_.

(*d*(*v*), *d*_2_(*v*))	Frequency
1, *k* + 1	$\frac{1}{3}\left[7^{r}\left(3k+1\right)2+4\right]$
*k* + 2, *k* + 1	$\frac{1}{3}\left[7^{r-1}\left(3k+1\right)2+4\right]$
*k* + 2, *k* + 4	$\frac{4}{3}\left[7^{r}-1\right]$
4, 4*k* + 4	$\frac{1}{3}\left[7^{r-1}\left(3k+1\right)-1\right]$

**Theorem 6.6**. *The First Leap Hyper Zagreb Index of Fractal tree F*_*r*_ *is denoted by HLM*_1_(*F*_*r*_) *and is given as*

$$\begin{array}{c} HLM_{1}\left(F_{r}\right)=7^{r-1}\left(24k^{3}+1328k+1338\right)+7^{r}\left(56k^{2}\right)-28k^{2}-144k-1880. \end{array}$$

*Proof*. We use Table 6 as follows.

$$\begin{array}{ccc} HLM_{1}\left(F_{r}\right) & = & \sum_{j=1}^{n}{\left[d_{2}\left(u_{j}\left(F_{r}\right)\right)+d_{2}\left(v_{j}\left(F_{r}\right)\right)\right]}^{2}\\ & = & {\left[\left(k+1\right)+\left(k+1\right)\right]}^{2}\left[7^{r-1}\left(3k+1\right)2+4\right]\\ & & +{\left[\left(k+1\right)+\left(k+4\right)\right]}^{2}\frac{1}{3}\left[10\left(7^{r}\right)-4\right]\\ & & +{\left[\left(k+4\right)+\left(k+4\right)\right]}^{2}\frac{1}{3}\left[7^{r}-4\right]+{\left[\left(k+4\right)+\left(4k+4\right)\right]}^{2}\frac{4}{3}\left[7^{r}-1\right]\\ HLM_{1}\left(F_{r}\right) & = & 7^{r-1}\left(24k^{3}+1328k+1338\right)+7^{r}\left(56k^{2}\right)-28k^{2}-144k-188. \end{array}$$

By Eq (17) and Table 6, we get

$$\begin{array}{ccc} E_{HLM_{1}}\left(F_{r}\right) & = & \text{log}[7^{r-1}\left(24k^{3}+1328k+1338\right)+7^{r}\left(56k^{2}\right)-28k^{2}-144k-188]\\ & & -\frac{\left[7^{r-1}\left(3k+1\right)2+4\right]\left(4k^{2}+8k+4\right)\text{log}\left(4k^{2}+8k+4\right)}{7^{r-1}\left(24k^{3}+1328k+1338\right)+7^{r}\left(56k^{2}\right)-28k^{2}-144k-188}\\ & & -\frac{\left[\frac{1}{3}\left(10\left(7^{r}\right)-4\right)\right]\left(4k^{2}+20k+25\right)\text{log}\left(4k^{2}+20k+25\right)}{7^{r-1}\left(24k^{3}+1328k+1338\right)+7^{r}\left(56k^{2}\right)-28k^{2}-144k-188}\\ & & -\frac{\left[\frac{1}{3}\left(7^{r}-4\right)\right]\left(4k^{2}+32k+64\right)\text{log}\left(4k^{2}+32k+64\right)}{7^{r-1}\left(24k^{3}+1328k+1338\right)+7^{r}\left(56k^{2}\right)-28k^{2}-144k-188}\\ & & -\frac{\left[\frac{4}{3}\left(7^{r}-1\right)\right]\left(25k^{2}+80k+64\right)\text{log}\left(25k^{2}+80k+64\right)}{7^{r-1}\left(24k^{3}+1328k+1338\right)+7^{r}\left(56k^{2}\right)-28k^{2}-144k-188}. \end{array}$$

**Theorem 6.7**. *The second Leap Hyper Zagreb Index of Fractal tree F*_*r*_ *is denoted by HLM*_2_(*F*_*r*_) *and is given as*

$$\begin{array}{ccc} HLM_{2}\left(F_{r}\right) & = & 7^{r-1}\left(6k^{5}+201k^{4}+1808k^{3}+5958k^{2}+3362\right)+7^{r}\left(1074k\right)\\ & & -2(10k^{4}+232k^{3}+852k^{2}+1232k+700). \end{array}$$

*Proof*. We use Table 6 as follows.

$$\begin{array}{ccc} HLM_{2}\left(F_{r}\right) & = & \sum_{j=1}^{n}{\left[d_{2}\left(u_{j}\left(F_{r}\right)\right)d_{2}\left(v_{j}\left(F_{r}\right)\right)\right]}^{2}\\ & = & {\left[\left(k+1\right)\left(k+1\right)\right]}^{2}\left[7^{r-1}\left(3k+1\right)2+4\right]\\ & & +{\left[\left(k+1\right)\left(k+4\right)\right]}^{2}\frac{1}{3}\left[10\left(7^{r}\right)-4\right]\\ & & +{\left[\left(k+4\right)\left(k+4\right)\right]}^{2}\frac{1}{3}\left[7^{r}-4\right]\\ & & +{\left[\left(k+4\right)\left(4k+4\right)\right]}^{2}\frac{4}{3}\left[7^{r}-1\right]\\ HLM_{2}\left(F_{r}\right) & = & 7^{r-1}\left(6k^{5}+201k^{4}+1808k^{3}+5958k^{2}+3362\right)+7^{r}\left(1074k\right)\\ & & -2(10k^{4}+232k^{3}+852k^{2}+1232k+700). \end{array}$$

The Eq (17) and Table 6 gives

$$\begin{array}{ccc} E_{HLM_{2}}\left(F_{r}\right) & = & \text{log}[7^{r-1}\left(6k^{5}+201k^{4}+1808k^{3}+5958k^{2}+3362\right)+7^{r}\left(1074k\right)\\ & & -2(10k^{4}+232k^{3}+852k^{2}+1232k+700)]\\ & & -\frac{\left[7^{r-1}\left(3k+1\right)2+4\right]{\left(k+1\right)}^{4}\;\text{log}{\left(k+1\right)}^{4}}{HLM_{2}\left(F_{r}\right)}\\ & & -\frac{\frac{1}{3}\left(10\left(7^{r}\right)-4\right)\left({\left(k+4\right)}^{2}{\left(k+1\right)}^{2}\right)\text{log}\left({\left(k+4\right)}^{2}{\left(k+1\right)}^{2}\right)}{HLM_{2}\left(F_{r}\right)}\\ & & -\frac{\left[\frac{1}{3}\left(7^{r}-4\right)\right]{\left(k+4\right)}^{4}\;\text{log}{\left(k+4\right)}^{4}}{HLM_{2}\left(F_{r}\right)}\\ & & -\frac{\frac{4}{3}\left(7^{r}-1\right)\left(16{\left(k+4\right)}^{2}{\left(k+1\right)}^{2}\right)\text{log}\left(16{\left(k+4\right)}^{2}{\left(k+1\right)}^{2}\right)}{HLM_{2}\left(F_{r}\right)}. \end{array}$$

### 6.3 Forgotten coindex of *F*_*r*_

In this section we are going to commute Forgotten Co-index of *F*_*r*_. We use Lemma 1.1 and Theorem 2.1 in [20] for an alternating formula of F-co-index.

$$\begin{array}{ccc} N_{v} & = & n-1-d_{v}\\ \bar{F}\left(G\right) & = & \sum_{v\in V(G)}d_{v}^{2}\times N_{v}. \end{array}$$

**Theorem 6.8**. *The Forgotten Co-index of fractal Tree dendrimer is denoted by* $\bar{F}\left(F_{r}\right)$ *and is given as*

$$\begin{array}{c} \bar{F}\left(F_{r}\right)=49^{r}\left[4k^{3}+26k^{2}+58k+42\right]-7^{r}\left[2k^{3}+12k^{2}+34k+50\right]+20. \end{array}$$

*Proof*. We use Table 8 as follows.

$$\begin{array}{ccc} \bar{F}\left(F_{r}\right) & = & \sum_{v\in V(G)}d_{v}^{2}\times N_{v}\\ & = & {\left(1\right)}^{2}\left[7^{r}\left(2k+3\right)-1\right]\left[\frac{1}{3}7^{r}\left(3k+1\right)2+\frac{4}{3}\right]\\ & & +{\left(4\right)}^{2}\left[7^{r}\left(2k+3\right)-4\right]\left[\frac{1}{3}\left(7^{r}-1\right)\right]+{\left(k+2\right)}^{2}\left[7^{r}\left(2k+3\right)-\left(k+2\right)\right]\left[2\left(7^{r}\right)\right]\\ \bar{F}\left(F_{r}\right) & = & 49^{r}\left[4k^{3}+26k^{2}+58k+42\right]-7^{r}\left[2k^{3}+12k^{2}+34k+50\right]+20. \end{array}$$

**Table 8:** The first degrees of end-vertices of edges in $\bar{F_{r}}$.

*d*(*v*)	Frequency	non adjacency
1	$\frac{1}{3}\left[7^{r}\left(3k+1\right)2+4\right]$	7^*r*^[2 *k* + 3] − 1
4	$\frac{1}{3}\left[7^{r}-1\right]$	7^*r*^[2 *k* + 3] − 4
*k* + 2	2[7^*r*^]	7^*r*^[2 *k* + 3] − (*k* + 2)

The Eq (17) and Table 8 gives

$$\begin{array}{ccc} E_{\bar{F}\left(F_{r}\right)} & = & \text{log}[49^{r}\left[4k^{3}+26k^{2}+58k+42\right]-7^{r}\left[2k^{3}+12k^{2}+34k+50\right]+20]\\ & & -\frac{[\frac{1}{3}\left(7^{r}\left(3k+1\right)2+4\right)]1\;\text{log}\;1}{49^{r}\left[4k^{3}+26k^{2}+58k+42\right]-7^{r}\left[2k^{3}+12k^{2}+34k+50\right]+20}\\ & & -\frac{[\frac{1}{3}\left(7^{r}-1\right)]16\;\text{log}\;16}{49^{r}\left[4k^{3}+26k^{2}+58k+42\right]-7^{r}\left[2k^{3}+12k^{2}+34k+50\right]+20}\\ & & -\frac{\left[2\left(7^{r}\right)\right]\left(k^{2}+4k+4\right)\text{log}\left(k^{2}+4k+4\right)}{49^{r}\left[4k^{3}+26k^{2}+58k+42\right]-7^{r}\left[2k^{3}+12k^{2}+34k+50\right]+20}. \end{array}$$

## 7. Structure of Cayley tree *C*_*m*,*n*_

A tree in which every non-leaf graph vertex has a fixed number of branches called Cayley tree, *m* is called an *m*-Cayley tree. The star graph is a unique *m*-Cayley tree with *m* + 1 nodes. Path graphs are 2-Cayley trees. Bathelallice is another name of cayley tree *C*_*m*,*n*_. For construction of Cayley tree *C*_*m*,*n*_, *m* ≥ 3, *n* ≥ 1, where *m* is number of nodes. It is necessary to mention that iterative method is adopted here, *C*_*m*,0_ is the first iteration. It consists of only one vertex.

**Fig 5:**
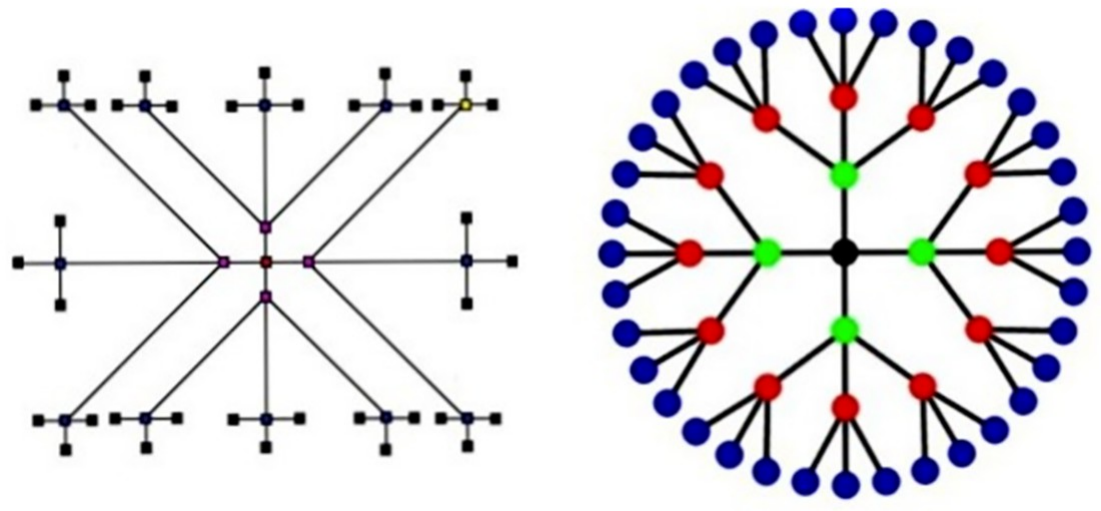
The graph of Cayley tree *C*_*m*,*n*_.

Then connect number of *m* nodes to the central vertex to get *C*_*m*,1_. Now joining all pendent vertices of *C*_*m*,*n*_ by creating *n* − 1 nodes of *C*_*m*,*n*−1_ to get *C*_*m*,*n*_. The graph of *C*_*m*,*n*_ is shown in Fig 5.

The pendents in *C*_*m*,*n*_ are *m*(*m* − 1)^*n*−1^ and number of vertices of degree m are $2\sum_{j=1}^{n}{\left(m-1\right)}^{j-1}-{\left(m-1\right)}^{n-1}$. Based on Degree of end vertices *C*_*m*,*n*_ has two type of edges2$\sum_{j=1}^{n}{\left(m-1\right)}^{j-1}+{\left(m-1\right)}^{n}$ and $\text{m}\sum_{j=1}^{n}{\left(m-1\right)}^{j-1}$

## 8. First, second and third degree of vertices of *C*_*m*,*n*_

In this section, we are looking forward for first, second and third degrees of vertices of *C*_*m*,*n*_, where *C*_*m*,*n*_ is a tree-type dendrimer. The order and size of graph *C*_*m*,*n*_ is given in Table 9. The first, second and third degree partition of *C*_*m*,*n*_ are given in Tables 10–12. The edge partition of *C*_*m*,*n*_ with respect to first degrees (*d*(*u*), *d*(*v*)), second degrees (*d*_2_(*u*), *d*_2_(*v*)) and first-second degrees (*d*(*u*), *d*_2_(*v*)) are given in Tables 13–15. The degree partition of vertices in ${\bar{C}}_{m,n}$ is given in Table 16.

**Table 9:** The order and size of *C*_*m*,*n*_.

Order of *C*_*m*,*n*_	Size of *C*_*m*,*n*_
$2\sum_{j=1}^{n}{\left(m-1\right)}^{j-1}+{\left(m-1\right)}^{n}$	$m\sum_{j=1}^{n}{\left(m-1\right)}^{j-1}$

**Table 10:** The Cayley tree *C*_*m*,*n*_ vertex degree partition.

*d*(*v*)	Frequency
1	*m*(*m* − 1)^*n*−1^
*m*	$2\sum_{j=1}^{n}{\left(m-1\right)}^{j-1}-{\left(m-1\right)}^{n-1}$

**Table 11:** The 2^*nd*^ degree vertex partition of *C*_*m*,*n*_.

*d*_2_(*u*)	Frequency
*m* − 1	4, *m*^2^(*m* − 1)^*n*−2^
*m*(*m* − 1)	$0,2\sum_{j=1}^{n-1}{\left(m-1\right)}^{j-1}-{\left(m-1\right)}^{n-2}$

**Table 12:** The 3^*rd*^ degree vertex partition of *C*_*m*,*n*_.

*d*_3_(*u*)	Frequency
*m* − 1	0, 12, *m*(*m* − 1)^*n*−1^
(*m* − 1)^2^	0, 4, *m*^2^(*m* − 1)^*n*−3^
*m*(*m* − 1)^2^	$0,0,2\sum_{j=1}^{n-2}{\left(m-1\right)}^{j-1}-{\left(m-1\right)}^{n-3}$

**Table 13:** The edge partition of *C*_*m*,*n*_.

*d*(*u*)*d*(*v*)	Frequency
(1, *m*)	*m*(*m* − 1)^*n*−1^
(*m*, *m*)	$m\sum_{j=1}^{n}{\left(m-1\right)}^{j-1}-{\left(m-1\right)}^{n-1}$

**Table 14:** The 2^*nd*^ degree edge partition of *C*_*m*,*n*_.

*d*_2_(*u*), *d*_2_(*v*)	Frequency
*m* − 1, *m* − 1	0, *m*(*m* − 1)^*n*−1^
*m* − 1, *m*(*m* − 1)	0, *m*(*m* − 1)^*n*−2^
*m*(*m* − 1), *m*(*m* − 1)	$0,m(\sum_{j=1}^{n-1}{\left(m-1\right)}^{j-1}-{\left(m-1\right)}^{n-2})$

**Table 15:** The 1^*st*^ & 2^*nd*^ degree edge partition of *C*_*m*,*n*_.

*d*_1_(*u*), *d*_2_(*u*)	Frequency
1, *m* − 1	4, *m*(*m* − 1)^*n*−1^
*m*, *m* − 1	0, *m*(*m* − 1)^*n*−2^
*m*, *m*(*m* − 1)	$0,2\sum_{j=1}^{n-1}{\left(m-1\right)}^{j-1}-{\left(m-1\right)}^{n-2}$

### 8.1 Wiener-type indices for *C*_*m*,*n*_

In this section we will discuss Wiener index of *C*_*m*,*n*_ with the help of table and basic results.

**Theorem 8.1**. *The Wiener index of Graph C*_*m*,*n*_ *is denoted by W*(*C*_*m*,*n*_) *and is given as*.

$$\begin{array}{c} W\left(C_{m,n}\right)=m\sum_{j=1}^{n}{\left(m-1\right)}^{j-1}. \end{array}$$

*Proof*. We use Table 10 as follows.

$$\begin{array}{ccc} W(C_{m,n}) & = & \frac{1}{2}\sum_{u\in V(C_{m,n})}d\left(v_{j}\left(C_{m,n}\right)\right)\\ & = & \frac{1}{2}{[\left(1\right)m\left(m-1\right)}^{n-1}+\left(m\right)\left[2\sum_{j=1}^{n}{\left(m-1\right)}^{j-1}-{\left(m-1\right)}^{n-1}\right]\\ W(C_{m,n}) & = & m\sum_{j=1}^{n}{\left(m-1\right)}^{j-1}. \end{array}$$

The Eq (17) and Table 10 gives

$$\begin{array}{ccc} E_{W}\left(C_{m,n}\right) & = & \text{log}[m\sum_{j=1}^{n}{\left(m-1\right)}^{j-1}]\\ & & -\frac{m{\left(m-1\right)}^{n-1}\left(0.5\right)\text{log}\left(0.5\right)}{m\sum_{j=1}^{n}{\left(m-1\right)}^{j-1}}\\ & & -\frac{\left[2\sum_{j=1}^{n}{\left(m-1\right)}^{j-1}-{\left(m-1\right)}^{n-1}\right]\left(\frac{m}{2}\right)\text{log}\left(\frac{m}{2}\right)}{m\sum_{j=1}^{n}{\left(m-1\right)}^{j-1}}. \end{array}$$

In this section we will discuss Wiener polarity index of *C*_*m*,*n*_ with the help of table and basic results.

**Theorem 8.2**. *The Wiener polarity index of Graph C*_*m*,*n*_ *is denoted by W*_*p*_(*C*_*m*,*n*_) *and is given as*.

$$\begin{array}{c} W_{p}\left(C_{m,n}\right)=m{\left(m-1\right)}^{2}\sum_{j=1}^{n-2}{\left(m-1\right)}^{j-1}+m{\left(m-1\right)}^{n}. \end{array}$$

*Proof*. By using the values from Table 12, we get Case 1: When *n* = 1.

$$\begin{array}{ccc} W_{p}\left(C_{m,1}\right) & = & \frac{1}{2}\sum_{j=1}^{n}d_{3}\left(v_{j}\left(C_{m,1}\right)\right)\\ & = & \frac{1}{2}{[\left(m-1\right)\left(0\right)+\left(m-1\right)}^{2}\left(0\right)\\ & & +(m{\left(m-1\right)}^{2})\left(0\right)]\\ W_{p}\left(C_{m,1}\right) & = & 0. \end{array}$$

The value $E_{W_{p}}\left(C_{m,1}\right)$ is undefined. Case 2: When *n* = 2.

$$\begin{array}{ccc} W_{p}\left(C_{m,2}\right) & = & \frac{1}{2}\sum_{j=1}^{n}d_{3}\left(v_{j}\left(C_{m,2}\right)\right)\\ & = & \frac{1}{2}{[\left(m-1\right)\left(12\right)+\left(m-1\right)}^{2}\left(4\right)\\ & & +(m{\left(m-1\right)}^{2})\left(0\right)]\\ W_{p}\left(C_{m,2}\right) & = & (2m^{2}+2m-4). \end{array}$$

The Eq (17) and Table 12 gives

$$\begin{array}{ccc} E_{W_{p}}\left(C_{m,2}\right) & = & \text{log}[\left(2m^{2}+2m-4\right)]\\ & & -\frac{\left(12\right)\left(\frac{\left(m-1\right)}{2}\right)\text{log}\left(\frac{\left(m-1\right)}{2}\right)}{\left(2m^{2}+2m-4\right)}\\ & & -\frac{\left(4\right)\left(\frac{{\left(m-1\right)}^{2}}{2}\right)\text{log}\left(\frac{{\left(m-1\right)}^{2}}{2}\right)}{\left(2m^{2}+2m-4\right)}. \end{array}$$

Case 3: When *n* ≥ 3.

$$\begin{array}{ccc} W_{p}\left(C_{m,n}\right) & = & \frac{1}{2}\sum_{j=1}^{n}d_{3}\left(v_{j}\left(C_{m,n}\right)\right)\\ & = & \frac{1}{2}[\left(m-1\right)\left(m{\left(m-1\right)}^{n-1}\right)+{\left(m-1\right)}^{2}\left(m^{2}{\left(m-1\right)}^{n-3}\right)\\ & & +\left(m{\left(m-1\right)}^{2}\right)\left(2\sum_{j=1}^{n-2}{\left(m-1\right)}^{j-1}-{\left(m-1\right)}^{n-3}\right)]\\ W_{p}\left(C_{m,n}\right) & = & m{\left(m-1\right)}^{2}\sum_{j=1}^{n-2}{\left(m-1\right)}^{j-1}+m{\left(m-1\right)}^{n}. \end{array}$$

The Eq (17) and Table 12 gives

$$\begin{array}{ccc} E_{W_{p}}\left(C_{m,n}\right) & = & \text{log}[m{\left(m-1\right)}^{2}\sum_{j=1}^{n-2}{\left(m-1\right)}^{j-1}+m{\left(m-1\right)}^{n}]\\ & & -\frac{\left(m{\left(m-1\right)}^{n-1}\right)\left(\frac{\left(m-1\right)}{2}\right)\text{log}\left(\frac{\left(m-1\right)}{2}\right)}{m{\left(m-1\right)}^{2}\sum_{j=1}^{n-2}{\left(m-1\right)}^{j-1}+m{\left(m-1\right)}^{n}}\\ & & -\frac{\left(m^{2}{\left(m-1\right)}^{n-3}\right)\left(\frac{{\left(m-1\right)}^{2}}{2}\right)\text{log}\left(\frac{{\left(m-1\right)}^{2}}{2}\right)}{m{\left(m-1\right)}^{2}\sum_{j=1}^{n-2}{\left(m-1\right)}^{j-1}+m{\left(m-1\right)}^{n}}\\ & & -\frac{\left(2\sum_{j=1}^{n-2}{\left(m-1\right)}^{j-1}-{\left(m-1\right)}^{n-3}\right)\left(\frac{\left(m{\left(m-1\right)}^{2}\right)}{2}\right)\text{log}\left(\frac{\left(m{\left(m-1\right)}^{2}\right)}{2}\right)}{m{\left(m-1\right)}^{2}\sum_{j=1}^{n-2}{\left(m-1\right)}^{j-1}+m{\left(m-1\right)}^{n}}. \end{array}$$

which completes the proof.

### 8.2 Zagreb-type indices of *C*_*m*,*n*_

In the very first stage we are going to calculate the leap Zagreb indices of first second and third versions for *C*_*m*,*n*_. After that we will discuss other indices.

**Theorem 8.3**. *The First Leap Zagreb Index of Cayley tree C*_*m*,*n*_ *is denoted by LM*_1_(*C*_*m*,*n*_) *and is given as*

$$\begin{array}{c} LM_{1}\left(C_{m,n}\right)=2m^{2}{\left(m-1\right)}^{2}\sum_{j=1}^{n-1}{\left(m-1\right)}^{j-1}. \end{array}$$

*Proof*. We use Table 11 as follows.

Case 1: When *n* = 1.

$$\begin{array}{ccc} LM_{1}\left(C_{m,1}\right) & = & \sum_{j=1}^{n}d_{2}^{2}\left(v_{j}\left(C_{m,1}\right)\right)\\ & = & \left(m-1)[4]+m(m-1)[0\right]\\ LM_{1}\left(C_{m,1}\right) & = & 4m-4. \end{array}$$

The Eq (17) and Table 11 gives

$$\begin{array}{ccc} E_{LM_{1}}\left(C_{m,1}\right) & = & \text{log}[4m-4]\\ & & -\frac{\left(4)(m-1)\text{log}(m-1\right)}{4m-4}. \end{array}$$

Case 2: When *n* ≥ 2.

$$\begin{array}{ccc} LM_{1}\left(C_{m,n}\right) & = & \sum_{j=1}^{n}d_{2}^{2}\left(v_{j}\left(C_{m,n}\right)\right)\\ & = & {\left(m-1\right)}^{2}\left[m^{2}{\left(m-1\right)}^{n-2}\right]+m^{2}{\left(m-1\right)}^{2}[2\sum_{j=1}^{n-1}{\left(m-1\right)}^{j-1}\\ & & -{\left(m-1\right)}^{n-2}]\\ LM_{1}\left(C_{m,n}\right) & = & 2m^{2}{\left(m-1\right)}^{2}\sum_{j=1}^{n-1}{\left(m-1\right)}^{j-1}. \end{array}$$

The Eq (17) and Table 11 gives

$$\begin{array}{ccc} E_{LM_{1}}\left(C_{m,n}\right) & = & \text{log}[2m^{2}{\left(m-1\right)}^{2}\sum_{j=1}^{n-1}{\left(m-1\right)}^{j-1}]\\ & & -\frac{\left[m^{2}{\left(m-1\right)}^{n-2}\right]\left(m^{2}-2m+1\right)\text{log}\left(m^{2}-2m+1\right)}{2m^{2}{\left(m-1\right)}^{2}\sum_{j=1}^{n-1}{\left(m-1\right)}^{j-1}}\\ & & -[2\sum_{j=1}^{n-1}{\left(m-1\right)}^{j-1}-{\left(m-1\right)}^{n-2}]\\ & & \times \frac{\left(m^{3}-2m^{2}+m\right)\text{log}\left(m^{3}-2m^{2}+m\right)}{2m^{2}{\left(m-1\right)}^{2}\sum_{j=1}^{n-1}{\left(m-1\right)}^{j-1}}. \end{array}$$

**Theorem 8.4**. *The Second Leap Zagreb Index of Cayley tree C*_*m*,*n*_ *is denoted by LM*_2_(*C*_*m*,*n*_) *and is given as*

$$\begin{array}{c} LM_{2}\left(C_{m,n}\right)=m^{3}{\left(m-1\right)}^{2}\sum_{j=1}^{n-1}{\left(m-1\right)}^{j-1}+m{\left(m-1\right)}^{n+1}. \end{array}$$

*Proof*. We use Table 14 as follows.

Case 1: When *n* = 1.

$$\begin{array}{ccc} LM_{2}\left(C_{m,1}\right) & = & \sum_{j=1}^{n}d_{2}\left(u_{j}\left(C_{m,1}\right)\right)d_{2}\left(v_{j}\left(C_{m,1}\right)\right)\\ & = & \left(m-1)(m-1)[0]+(m-1)m(m-1)[0\right]\\ & & +m(m-1)m(m-1)[0]\\ LM_{2}\left(C_{m,1}\right) & = & 0. \end{array}$$

The entropy is undefined for *n* = 1

Case 2: When *n* ≥ 2.

$$\begin{array}{ccc} LM_{2}\left(C_{m,n}\right) & = & \sum_{j=1}^{n}d_{2}\left(u_{j}\left(C_{m,n}\right)\right)d_{2}\left(v_{j}\left(C_{m,n}\right)\right)\\ & = & \left(m-1\right)\left(m-1\right)[m{\left(m-1\right)}^{n-1}]\\ & & +\left(m-1\right)m\left(m-1\right)[m{\left(m-1\right)}^{n-2}]\\ & & +m\left(m-1\right)m\left(m-1\right)[m\left(\sum_{j=1}^{n-1}{\left(m-1\right)}^{j-1}-{\left(m-1\right)}^{n-2}\right)]\\ LM_{2}\left(C_{m,n}\right) & = & m^{3}{\left(m-1\right)}^{2}\sum_{j=1}^{n-1}{\left(m-1\right)}^{j-1}+m{\left(m-1\right)}^{n+1}. \end{array}$$

The Eq (17) and Table 14 gives

$$\begin{array}{ccc} E_{LM_{2}}\left(C_{m,n}\right) & = & \text{log}[m^{3}{\left(m-1\right)}^{2}\sum_{j=1}^{n-1}{\left(m-1\right)}^{j-1}+m{\left(m-1\right)}^{n+1}]\\ & & -\frac{\left[m{\left(m-1\right)}^{n-1}\right]\left(m^{2}-2m+1\right)\text{log}\left(m^{2}-2m+1\right)}{m^{3}{\left(m-1\right)}^{2}\sum_{j=1}^{n-1}{\left(m-1\right)}^{j-1}+m{\left(m-1\right)}^{n+1}}\\ & & -\frac{\left[m{\left(m-1\right)}^{n-2}\right]\left(m^{3}-2m^{2}+m\right)\text{log}\left(m^{3}-2m^{2}+m\right)}{m^{3}{\left(m-1\right)}^{2}\sum_{j=1}^{n-1}{\left(m-1\right)}^{j-1}+m{\left(m-1\right)}^{n+1}}\\ & & -[m\left(\sum_{j=1}^{n-1}{\left(m-1\right)}^{j-1}-{\left(m-1\right)}^{n-2}\right)]\\ & & \times \frac{\left(m^{4}-2m^{3}+m^{2}\right)\text{log}\left(m^{4}-2m^{3}+m^{2}\right)}{m^{3}{\left(m-1\right)}^{2}\sum_{j=1}^{n-1}{\left(m-1\right)}^{j-1}+m{\left(m-1\right)}^{n+1}}. \end{array}$$

**Theorem 8.5**. *The Third Leap Zagreb Index of Cayley tree C*_*m*,*n*_ *is denoted by LM*_3_(*C*_*m*,*n*_) *and is given as*

$$\begin{array}{c} LM_{3}\left(C_{m,n}\right)=2m^{2}\left(m-1\right)\sum_{j=1}^{n-1}{\left(m-1\right)}^{j-1}+m{\left(m-1\right)}^{n}. \end{array}$$

*Proof*. We use Table 15 as follows.

Case 1: When *n* = 1.

$$\begin{array}{ccc} LM_{3}\left(C_{m,1}\right) & = & \sum_{j=1}^{n}d_{1}\left(v_{j}\left(C_{m,1}\right)\right)d_{2}\left(v_{j}\left(C_{m,1}\right)\right)\\ & = & \left(1)(m-1)[4\right]\\ & & +(m)(m-1)[0]\\ & & +(m)m(m-1)[0].\\ LM_{3}\left(C_{m,1}\right) & = & 4m-4. \end{array}$$

The Eq (17) and Table 15 gives

$$\begin{array}{ccc} E_{LM_{3}}\left(C_{m,1}\right) & = & \text{log}[4m-4]\\ & & -\frac{\left[4](m-1)\text{log}(m-1\right)}{4m-4}. \end{array}$$

Case 2: When *n* ≥ 2.

$$\begin{array}{ccc} LM_{3}\left(C_{m,n}\right) & = & \sum_{j=1}^{n}d_{1}\left(v_{j}\left(C_{m,n}\right)\right)d_{2}\left(v_{j}\left(C_{m,n}\right)\right)\\ & = & \left(1\right)\left(m-1\right)[m{\left(m-1\right)}^{n-1}]\\ & & +\left(m\right)\left(m-1\right)[m{\left(m-1\right)}^{n-2}]\\ & & +\left(m\right)m\left(m-1\right)[2\sum_{j=1}^{n-1}{\left(m-1\right)}^{j-1}-{\left(m-1\right)}^{n-2}].\\ LM_{3}\left(C_{m,n}\right) & = & 2m^{2}\left(m-1\right)\sum_{j=1}^{n-1}{\left(m-1\right)}^{j-1}+m{\left(m-1\right)}^{n}. \end{array}$$

The Eq (17) and Table 15 gives

$$\begin{array}{ccc} E_{LM_{3}}\left(C_{m,n}\right) & = & \text{log}[2m^{2}\left(m-1\right)\sum_{j=1}^{n-1}{\left(m-1\right)}^{j-1}+m{\left(m-1\right)}^{n}]\\ & & -\frac{[m{\left(m-1\right)}^{n-1}]\left(m-1\right)\text{log}\left(m-1\right)}{2m^{2}\left(m-1\right)\sum_{j=1}^{n-1}{\left(m-1\right)}^{j-1}+m{\left(m-1\right)}^{n}}\\ & & -\frac{\left[m{\left(m-1\right)}^{n-2}\right]\left(m^{2}-m\right)\text{log}\left(m^{2}-m\right)}{2m^{2}\left(m-1\right)\sum_{j=1}^{n-1}{\left(m-1\right)}^{j-1}+m{\left(m-1\right)}^{n}}\\ & & -[2\sum_{j=1}^{n-1}{\left(m-1\right)}^{j-1}-{\left(m-1\right)}^{n-2}]\\ & & \times \frac{\left(m^{3}-m^{2}\right)\text{log}\left(m^{3}-m^{2}\right)}{2m^{2}\left(m-1\right)\sum_{j=1}^{n-1}{\left(m-1\right)}^{j-1}+m{\left(m-1\right)}^{n}}. \end{array}$$

**Theorem 8.6**. *The First Leap Hyper Zagreb Index of Cayley tree C*_*m*,*n*_ *is denoted by HLM*_1_(*C*_*m*,*n*_) *and is given as*

$$\begin{array}{c} HLM_{1}\left(C_{m,n}\right)=4m^{3}{\left(m-1\right)}^{2}\sum_{j=1}^{n-1}{\left(m-1\right)}^{j-1}-3m{\left(m-1\right)}^{2}{\left(m-1\right)}^{n}. \end{array}$$

*Proof*. We use Table 15 as follows.

Case 1: When *n* = 1.

$$\begin{array}{ccc} HLM_{1}\left(C_{m,1}\right) & = & \sum_{uv\in E(C_{m,1})}{\left[d_{2}\left(u_{j}\left(C_{m,1}\right)\right)+d_{2}\left(v_{j}\left(C_{m,1}\right)\right)\right]}^{2}\\ & = & 2{\left(m-1\right)}^{2}\left[0\right]+{\left(m^{2}-1\right)}^{2}\left[0\right]\\ & & +4m^{2}{\left(m-1\right)}^{2}\left[0\right]\\ HLM_{1}\left(C_{m,1}\right) & = & 0. \end{array}$$

The entropy is undefined for *n* = 1.

Case 2: When *n* ≥ 2.

$$\begin{array}{ccc} HLM_{1}\left(C_{m,n}\right) & = & \sum_{uv\in E(C_{m,n})}{\left[d_{2}\left(u_{j}\left(C_{m,n}\right)\right)+d_{2}\left(v_{j}\left(C_{m,n}\right)\right)\right]}^{2}\\ & = & 4{\left(m-1\right)}^{2}\left[m{\left(m-1\right)}^{n-1}\right]\\ & & +{\left(m^{2}-1\right)}^{2}\left[m{\left(m-1\right)}^{n-2}\right]\\ & & +4m^{2}{\left(m-1\right)}^{2}\left[m\left(\sum_{j=1}^{n-1}{\left(m-1\right)}^{j-1}-{\left(m-1\right)}^{n-2}\right)\right]\\ HLM_{1}\left(C_{m,n}\right) & = & 4m^{3}{\left(m-1\right)}^{2}\sum_{j=1}^{n-1}{\left(m-1\right)}^{j-1}-3m{\left(m-1\right)}^{2}{\left(m-1\right)}^{n}. \end{array}$$

The Eq (17) and Table 13 gives

$$\begin{array}{ccc} E_{HLM_{1}}\left(C_{m,n}\right) & = & \text{log}[4m^{3}{\left(m-1\right)}^{2}\sum_{j=1}^{n-1}{\left(m-1\right)}^{j-1}-3m{\left(m-1\right)}^{2}{\left(m-1\right)}^{n}]\\ & & -\frac{\left[m{\left(m-1\right)}^{n-1}\right]\left(4m^{2}-8m+4\right)\text{log}\left(4m^{2}-8m+4\right)}{4m^{3}{\left(m-1\right)}^{2}\sum_{j=1}^{n-1}{\left(m-1\right)}^{j-1}-3m{\left(m-1\right)}^{2}{\left(m-1\right)}^{n}}\\ & & -\frac{\left[m{\left(m-1\right)}^{n-2}\right]\left(m^{4}-2m^{2}+1\right)\text{log}\left(m^{4}-2m^{2}+1\right)}{4m^{3}{\left(m-1\right)}^{2}\sum_{j=1}^{n-1}{\left(m-1\right)}^{j-1}-3m{\left(m-1\right)}^{2}{\left(m-1\right)}^{n}}\\ & & -[m\left(\sum_{j=1}^{n-1}{\left(m-1\right)}^{j-1}-{\left(m-1\right)}^{n-2}\right)]\\ & & \times \frac{\left(4m^{4}-8m^{3}+4m^{2}\right)\text{log}\left(4m^{4}-8m^{3}+4m^{2}\right)}{4m^{3}{\left(m-1\right)}^{2}\sum_{j=1}^{n-1}{\left(m-1\right)}^{j-1}-3m{\left(m-1\right)}^{2}{\left(m-1\right)}^{n}}. \end{array}$$

**Theorem 8.7**. *The Second Leap Hyper Zagreb Index of Cayley tree C*_*m*,*n*_ *is denoted by HLM*_2_(*C*_*m*,*n*_) *and is given as*

$$\begin{array}{c} HLM_{2}\left(C_{m,n}\right)=m^{5}{\left(m-1\right)}^{4}\sum_{j=1}^{n-1}{\left(m-1\right)}^{j-1}-\left(m^{5}-m^{3}-m^{2}+m\right){\left(m-1\right)}^{n+2}. \end{array}$$

*Proof*. We use Table 15 as follows.

Case 1: When *n* = 1.

$$\begin{array}{ccc} HLM_{2}\left(C_{m,1}\right) & = & \sum_{uv\in E(C_{m,1})}{\left[d_{2}\left(u_{j}\left(C_{m,1}\right)\right)d_{2}\left(v_{j}\left(C_{m,1}\right)\right)\right]}^{2}\\ & = & {\left[{\left(m-1\right)}^{2}\right]}^{2}\left[0\right]+{\left[m{\left(m-1\right)}^{2}\right]}^{2}\left[0\right]\\ & & +{\left[m^{2}{\left(m-1\right)}^{2}\right]}^{2}\left[0\right]\\ HLM_{2}\left(C_{m,1}\right) & = & 0. \end{array}$$

The entropy is undefined for *n* = 1.

Case 2: When *n* ≥ 2.

$$\begin{array}{ccc} HLM_{2}\left(C_{m,n}\right) & = & \sum_{uv\in E(C_{m,2})}{\left[d_{2}\left(u_{j}\left(C_{m,n}\right)\right)d_{2}\left(v_{j}\left(C_{m,n}\right)\right)\right]}^{2}\\ & = & {\left[{\left(m-1\right)}^{2}\right]}^{2}\left[m{\left(m-1\right)}^{n-1}\right]\\ & & +{\left[m{\left(m-1\right)}^{2}\right]}^{2}\left[m{\left(m-1\right)}^{n-2}\right]\\ & & +{\left[m^{2}{\left(m-1\right)}^{2}\right]}^{2}\left[m\left(\sum_{j=1}^{n-1}{\left(m-1\right)}^{j-1}-{\left(m-1\right)}^{n-2}\right)\right]\\ HLM_{2}\left(C_{m,n}\right) & = & m^{5}{\left(m-1\right)}^{4}\sum_{j=1}^{n-1}{\left(m-1\right)}^{j-1}-\left(m^{5}-m^{3}-m^{2}+m\right){\left(m-1\right)}^{n+2}. \end{array}$$

The Eq (17) and Table 15 gives

$$\begin{array}{ccc} E_{HLM_{2}}\left(C_{m,n}\right) & = & \text{log}[m^{5}{\left(m-1\right)}^{4}\sum_{j=1}^{n-1}{\left(m-1\right)}^{j-1}\\ & & -\left(m^{5}-m^{3}-m^{2}+m\right){\left(m-1\right)}^{n+2}]\\ & & -\frac{\left[m{\left(m-1\right)}^{n-1}\right]{\left(m-1\right)}^{4}\;\text{log}{\left(m-1\right)}^{4}}{HLM_{2}\left(C_{m,n}\right)}\\ & & -\frac{\left[m{\left(m-1\right)}^{n-2}\right]\left(m^{2}{\left(m-1\right)}^{4}\right)\text{log}\left(m^{2}{\left(m-1\right)}^{4}\right)}{HLM_{2}\left(C_{m,n}\right)}\\ & & -[m\left(\sum_{j=1}^{n-1}{\left(m-1\right)}^{j-1}-{\left(m-1\right)}^{n-2}\right)]\\ & & \times \frac{\left(m^{4}{\left(m-1\right)}^{4}\right)\text{log}\left(m^{4}{\left(m-1\right)}^{4}\right)}{HLM_{2}\left(C_{m,n}\right)}. \end{array}$$

### 8.3 Forgotten coindex of *C*_*m*,*n*_

In this section we are going to commute Forgotten Co-index of *C*_*m*,*n*_. We use Lemma (1.1) and Theorem (2.1) in [20] for an alternating formula of the F-co-index.

$$\begin{array}{ccc} N_{v} & = & n-1-d_{v}\\ \bar{F}\left(G\right) & = & \sum_{v\in V(G)}d_{v}^{2}\times N_{v}. \end{array}$$

**Theorem 8.8**. *The Forgotten Co-index of Cayley Tree dendrimer is denoted by* $\bar{F}\left(C_{m,n}\right)$ *and is given as*

$$\begin{array}{ccc} \bar{F}\left(C_{m,n}\right) & = & 4m^{2}{\left[\sum_{j=1}^{n}{\left(m-1\right)}^{j-1}\right]}^{2}-2m\left(m^{2}+m-{\left(m-1\right)}^{n+1}\right)\left[\sum_{j=1}^{n}{\left(m-1\right)}^{j-1}\right]\\ & & -m[{\left(m-1\right)}^{2n}-\left(m+2\right){\left(m-1\right)}^{n}]. \end{array}$$

*Proof*. We use Table 16 as follows.

$$\begin{array}{ccc} \bar{F}\left(C_{m,n}\right) & = & \sum_{v\in V(G)}d_{v}^{2}\times N_{v}\\ & = & {\left(1\right)}^{2}\left[m{\left(m-1\right)}^{n-1}\right]\left[2\sum_{j=1}^{n}{\left(m-1\right)}^{j-1}+{\left(m-1\right)}^{n}-2\right]\\ & & +{\left(m\right)}^{2}\left[2\sum_{j=1}^{n}{\left(m-1\right)}^{j-1}-{\left(m-1\right)}^{n-1}\right][2\sum_{j=1}^{n}{\left(m-1\right)}^{j-1}\\ & & +{\left(m-1\right)}^{n}-\left(m+1\right)]\\ \bar{F}\left(C_{m,n}\right) & = & 4m^{2}{\left[\sum_{j=1}^{n}{\left(m-1\right)}^{j-1}\right]}^{2}-2m\left(m^{2}+m-{\left(m-1\right)}^{n+1}\right)\left[\sum_{j=1}^{n}{\left(m-1\right)}^{j-1}\right]\\ & & -m[{\left(m-1\right)}^{2n}-\left(m+2\right){\left(m-1\right)}^{n}]. \end{array}$$

**Table 16:** The degrees, frequencies & non-adjacencies in *C*_*m*,*n*_.

*d*(*v*)	Frequency	non-adjacency
1	*m*(*m* − 1)^*n*−1^	$2\sum_{j=1}^{n}{\left(m-1\right)}^{j-1}$ +(*m* − 1)^*n*^−2
*m*	$2\sum_{j=1}^{n}{\left(m-1\right)}^{j-1}-{\left(m-1\right)}^{n-1}$	$2\sum_{j=1}^{n}{\left(m-1\right)}^{j-1}$ + (*m* − 1)^*n*^ − (*m* + 1)

The Eq (17) and Table 16 gives

$$\begin{array}{ccc} E_{\bar{F}}\left(C_{m,n}\right) & = & \text{log}[4m^{2}{\left[\sum_{j=1}^{n}{\left(m-1\right)}^{j-1}\right]}^{2}-2m\left(m^{2}+m-{\left(m-1\right)}^{n+1}\right)\left[\sum_{j=1}^{n}{\left(m-1\right)}^{j-1}\right]\\ & & -m[{\left(m-1\right)}^{2n}-\left(m+2\right){\left(m-1\right)}^{n}]]\\ & & -\frac{[m{\left(m-1\right)}^{n-1}]1\;\text{log}\;1}{\bar{F}\left(C_{m,n}\right)}\\ & & -\frac{\left[2\sum_{j=1}^{n}{\left(m-1\right)}^{j-1}-{\left(m-1\right)}^{n-1}\right]\left(m^{2}\right)\text{log}\left(m^{2}\right)}{\bar{F}\left(C_{m,n}\right)}. \end{array}$$

## 9. Graphical comparisons of *F*_*r*_

In this section, the topological indices are being compared with the help of Fractal tree *F*_*r*_. Entropy is a structural descriptor that may easily be used to assess how complicated chemical systems are. To analyze algorithms and information flow, distance-based topological indices are often employed in computer science. Software engineering and pharmaceutical industries frequently employ degree-based entropy for a variety of purposes like melting point boiling points strain energy etc.

A variety of assessments, including those involving computer algorithms, information flow, melting points, and other topics, can be made using distance and degree-based indices. In this regard, they are rapid and have a clear decision-making procedure. Due to the complexity of molecular structures, it is possible to anticipate the most structural information by using a combination of molecular descriptors and associated entropy metrics.

**Fig 6:**
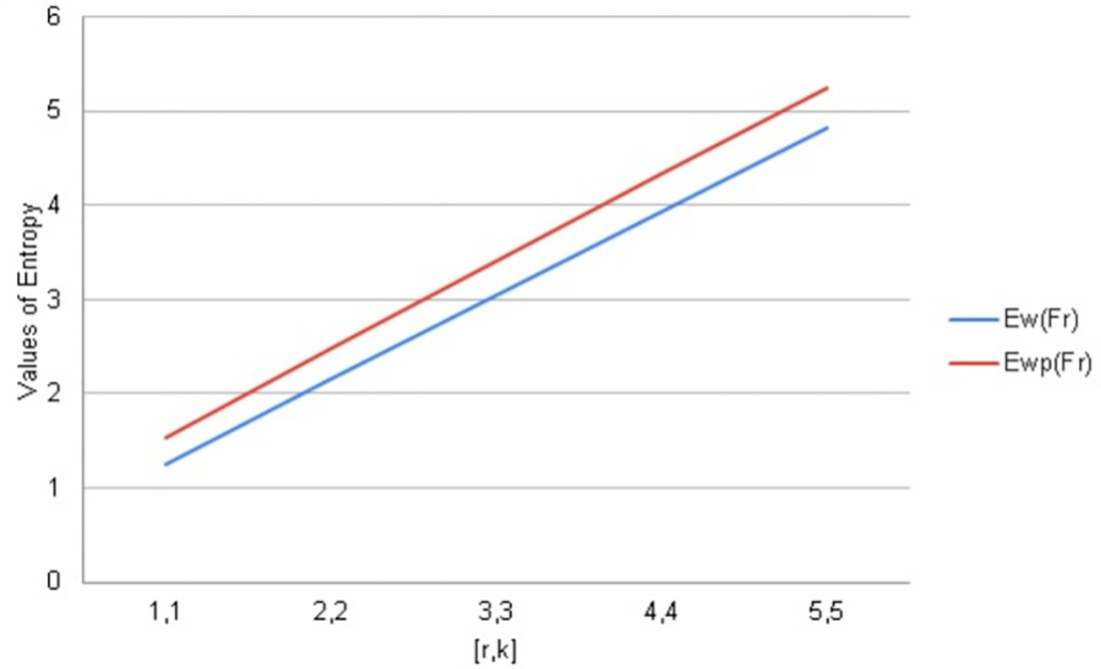
Graphical comparison of entropy for *F*_*r*_.

**Fig 7:**
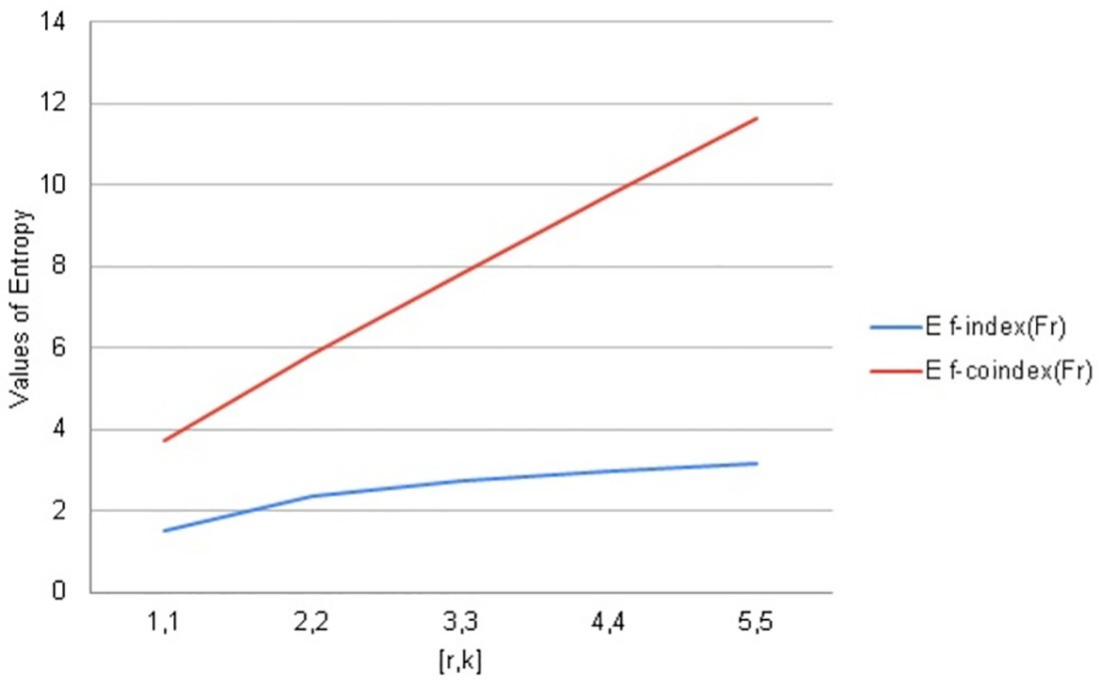
Graphical comparison of entropy for *F*_*r*_.

**Fig 8:**
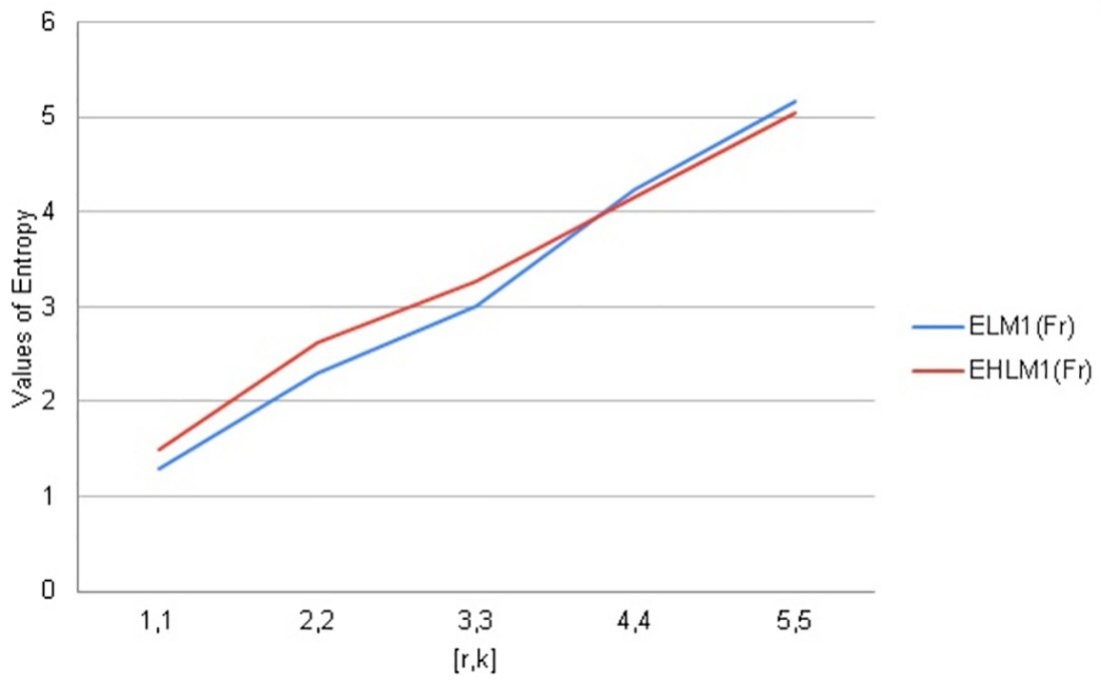
Graphical comparison of entropy for *F*_*r*_.

**Fig 9:**
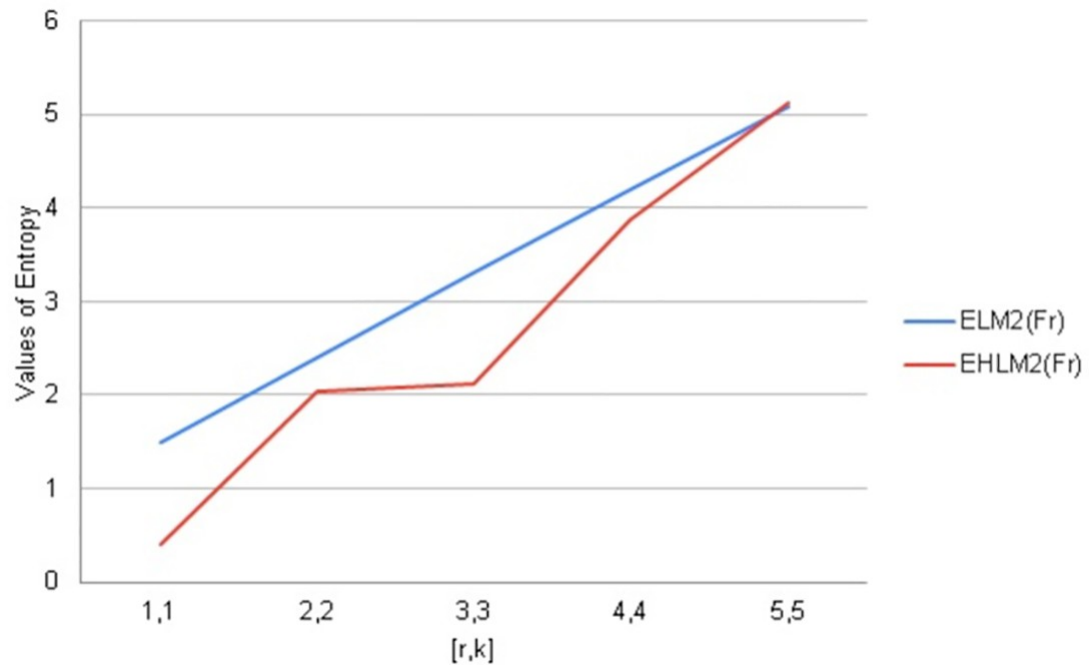
Graphical comparison of entropy for *F*_*r*_.

**Fig 10:**
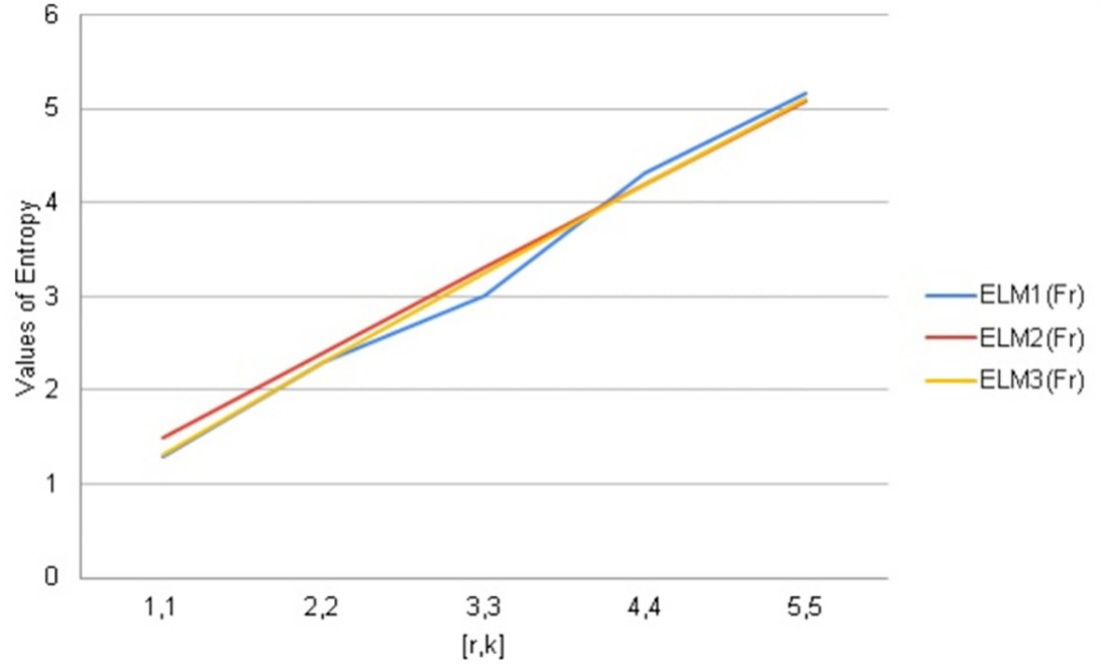
Graphical comparison of entropy for *F*_*r*_.

We have calculated arithmetic values of distance and degree-based topological indices entropies for fractal tree *F*_*r*_. We used Maple software and Online Excel Sheets for this purpose. For different values of [*r*, *k*] comparison diagrams and the data is presented in Tables 17–21. An increase in generation size resulted in a monotonically growing entropy function for the fractal tree *F*_*r*_. This behavior is shown in Figs 6–10.

**Table 17:** Wiener & Wiener polarity Entropy values for *F*_*r*_.

[*r*, *k*]	$ENT_{w_{p}}\left(F_{r}\right)$	*ENT*_*w*_(*F*_*r*_)
1, 1	1.2531	1.5299
2, 2	2.1580	2.4824
3, 3	3.0504	3.4165
4, 4	3.9352	4.3347
5, 5	4.8153	5.2418

**Table 18:** Forgotten & Forgotten co-index Entropy values for *F*_*r*_.

[*r*, *k*]	*ENT*_*F*_(*F*_*r*_)	$ENT_{\bar{F}}\left(F_{r}\right)$
1, 1	1.5085	3.7284
2, 2	2.3711	5.8401
3, 3	2.7293	7.8161
4, 4	2.9743	9.7365
5, 5	3.1626	11.6218

**Table 19:** First Leap Zagreb & First Leap Hyper Zagreb Entropy values for *F*_*r*_.

[*r*, *k*]	$ENT_{LM_{1}}\left(F_{r}\right)$	$ENT_{HLM_{1}}\left(F_{r}\right)$
1, 1	1.2864	1.5005
2, 2	2.2941	2.6292
3, 3	3.0037	3.2790
4, 4	4.2322	4.1638
5, 5	5.1733	5.04802

**Table 20:** 2^*nd*^ Leap Zagreb & 2^*nd*^ Leap Hyper Zagreb Entropy values for *F*_*r*_.

[*r*, *k*]	$ENT_{LM_{2}}\left(F_{r}\right)$	$ENT_{HLM_{2}}\left(F_{r}\right)$
1, 1	1.4881	0.4051
2, 2	2.4019	2.0363
3, 3	3.3003	2.1247
4, 4	4.1949	3.8664
5, 5	5.0862	5.12298

**Table 21:** Second Leap Zagreb & Second Leap Hyper Zagreb Entropy values for *F*_*r*_.

[*r*, *k*]	$ENT_{LM_{1}}\left(F_{r}\right)$	$ENT_{LM_{2}}\left(F_{r}\right)$	$ENT_{LM_{2}}\left(F_{r}\right)$
1, 1	1.2864	1.4881	1.3159
2, 2	2.2940	2.4019	2.3028
3, 3	3.0037	3.3003	3.2579
4, 4	4.2322	4.1949	4.1889
5, 5	5.1733	5.0862	5.1030

To compare how Entropy increases as the structure of *F*_*r*_ expands. Above graphical representation shows that Wiener index and Wiener polarity Entropy increases monotonically but showing similar increasing behavior.

To compare how Entropy increases as the structure of *F*_*r*_ expands. Above graphical representation shows that forgotten index and Forgotten co-index Entropy increases monotonically but forgotten co-index entropy increases more rapidly as *F*_*r*_ expands. We have taken the values of Forgottan index from the paper Imran et al., [45].

To compare how Entropy increases as the structure of *F*_*r*_ expands. Above graphical representation shows that First Leap Zagreb and First Leap Hyper Zagreb Entropy increases monotonically but in the beginning First Leap Hyper Zagreb entropy increases slightly more than First Leap Zagreb entropy. As *F*_*r*_ expands First Leap Zagreb entropy shows similar behavior for Leap Hyper Zagreb entropy.

To compare how Entropy increases as the structure of *F*_*r*_ expands. Above graphical representation shows that Second Leap Zagreb and Second Leap Hyper Zagreb Entropy increases monotonically but in the beginning First Leap Zagreb entropy increases slightly more than First Leap Hyper Zagreb entropy. As *F*_*r*_ expands First Leap Zagreb entropy increases rapidly but for Leap Hyper Zagreb entropy shows some times slightly little change in the behavior.

To compare how Entropy increases as the structure of *F*_*r*_ expands. Above graphical representation shows that First Second and third Leap Zagreb index Entropy increases monotonically but as *F*_*r*_ expands mostly all these entropies shows similar behavior but slightly different.

## 10. Graphical comparisons of *C*_*m*,*n*_

**Fig 11:**
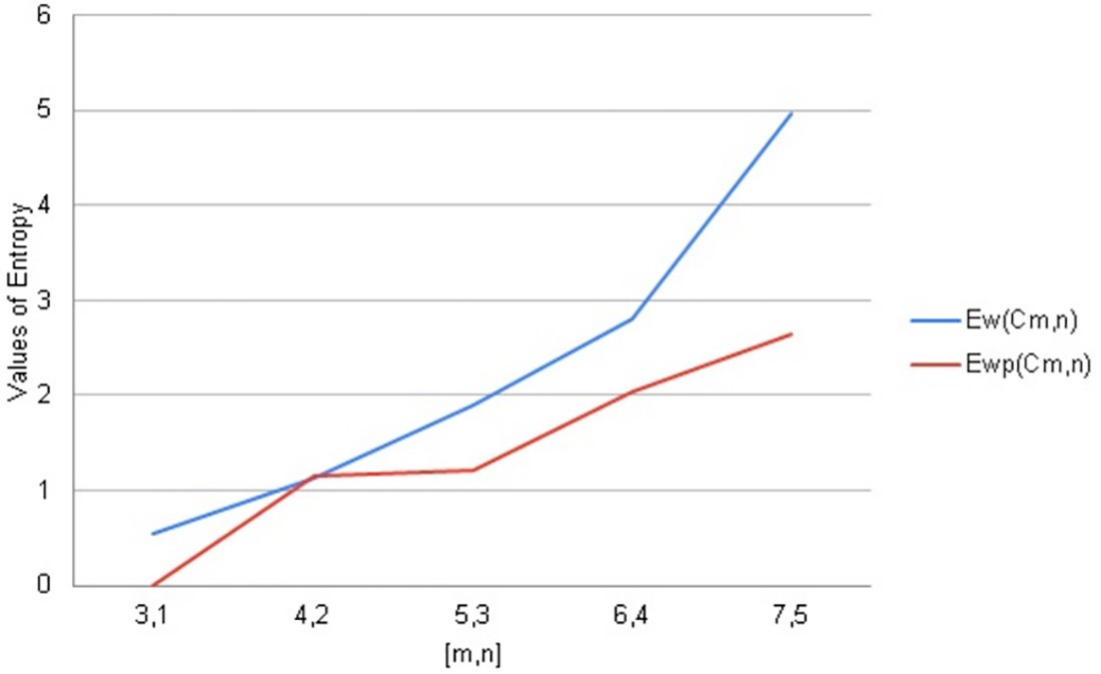
Graphical comparison of entropy for *C*_*m*,*n*_.

**Fig 12:**
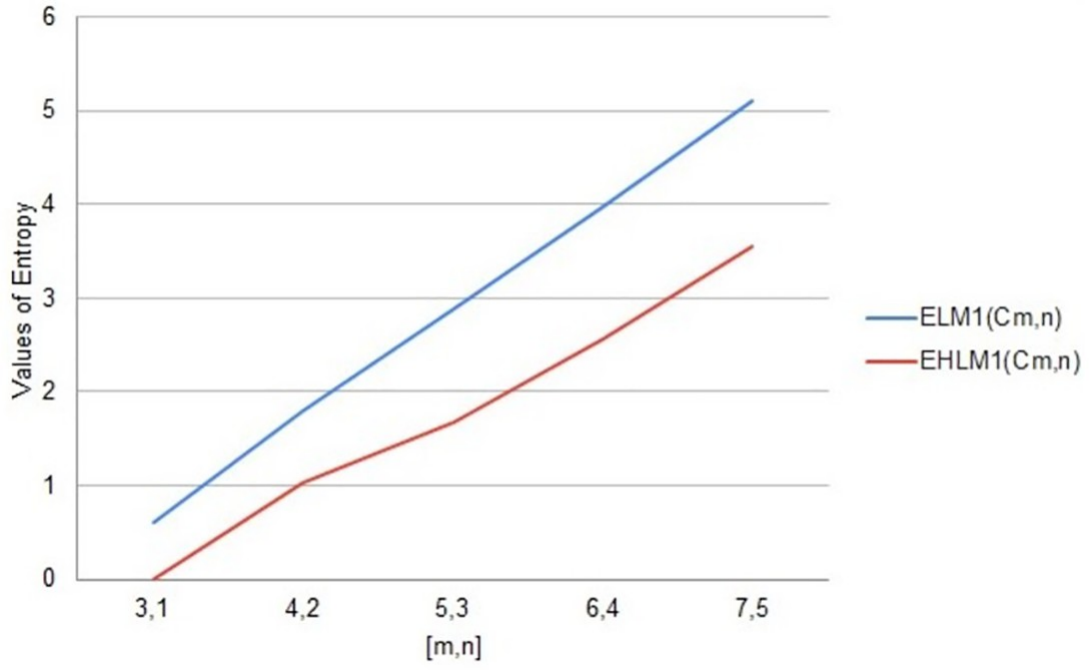
Graphical comparison of entropy for *C*_*m*,*n*_.

**Fig 13:**
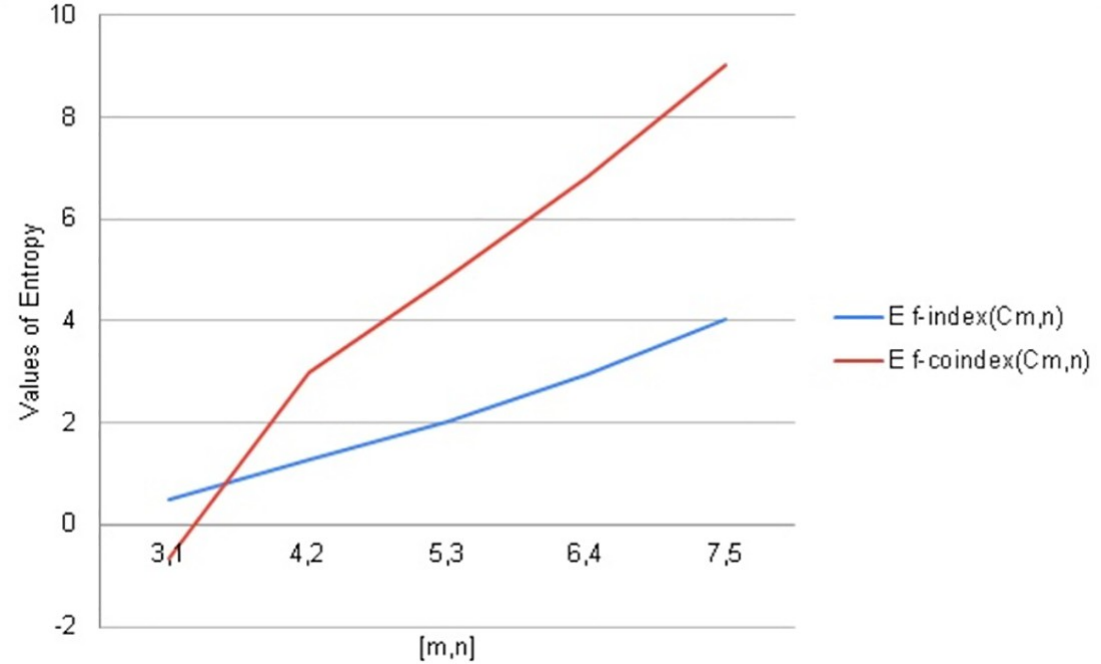
Graphical comparison of entropy for *C*_*m*,*n*_.

**Fig 14:**
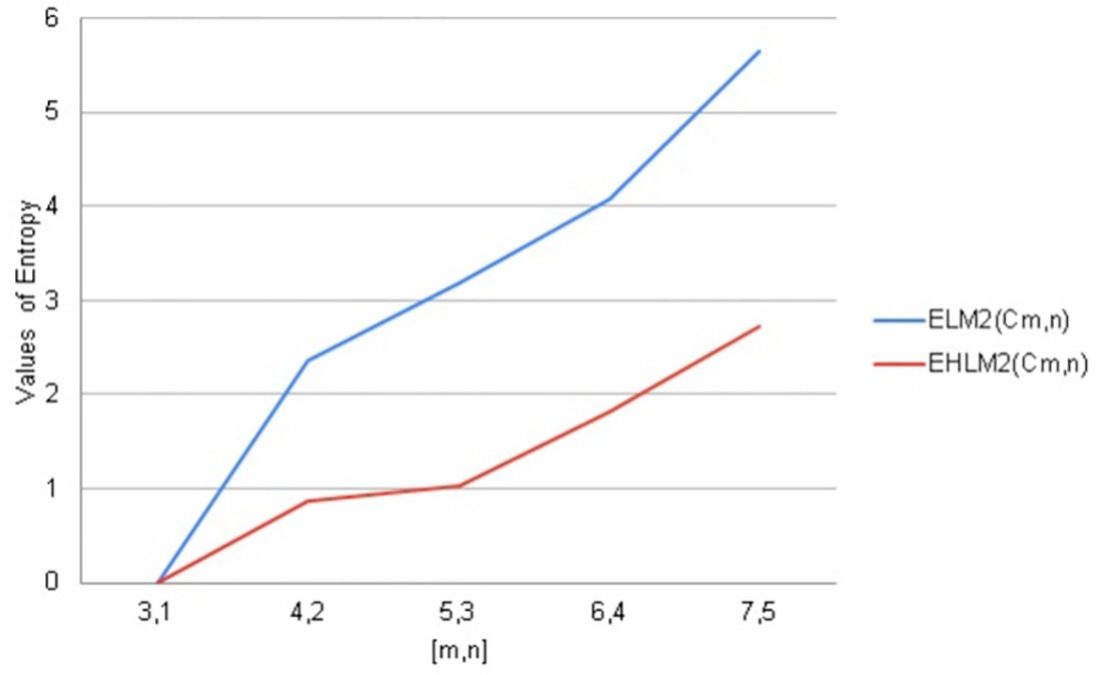
Graphical comparison of entropy for *C*_*m*,*n*_.

**Fig 15:**
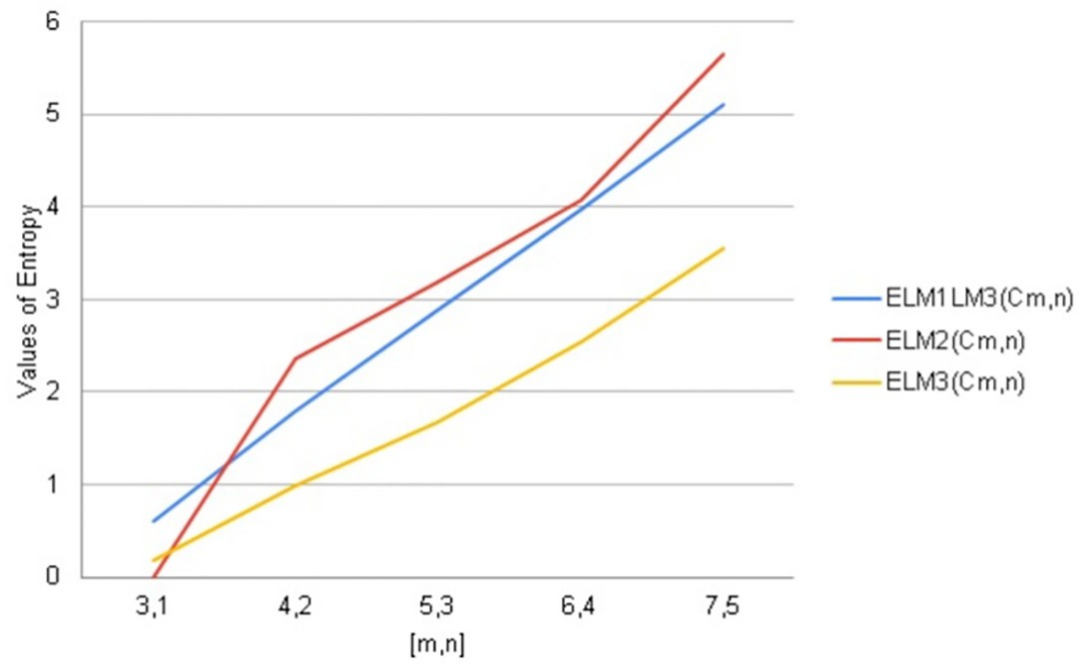
Graphical comparison of entropy for *C*_*m*,*n*_.

Entropy is a notion in science and a quantifiable physical characteristic that is frequently linked to a condition of disorder unpredictability, or uncertainty. arithmetic values of distance and degree-based topological indices entropies for Cayley tree *C*_*m*,*n*_. We used Maple software and Online Excel Sheets for this purpose. For different values of [*m*, *n*] comparison diagrams are given in Tables 22–26. An increase in generation size resulted in increasing values of entropy function monotonically for a Cayley tree type dendrimer. This increase is shown in Figs 11–15.

**Table 22:** Wiener & Wiener polarity Entropy values for *C*_*m*,*n*_.

[*m*, *n*]	$ENT_{w_{p}}\left(C_{m,n}\right)$	*ENT*_*w*_(*C*_*m*,*n*_)
3, 1	0.5395	0
4, 2	1.1289	1.1416
5, 3	1.8895	1.2096
6, 4	2.8059	2.0442
7, 5	4.9739	52.6346

**Table 23:** Forgotten & Forgotten co-index Entropy values for *C*_*m*,*n*_.

[*m*, *n*]	*ENT*_*F*_(*C*_*m*,*n*_	$ENT_{\bar{F}}\left(C_{m,n}\right)$
3, 1	0.4771	−0.6532
4, 2	1.2848	2.9724
5, 3	2.0009	4.8528
6, 4	2.9520	6.8412
7, 5	4.0290	9.0275

**Table 24:** First Leap Zagreb & First Leap Hyper Zagreb Entropy values for *C*_*m*,*n*_.

[*m*, *n*]	$ENT_{LM_{1}}\left(C_{m,n}\right)$	$ENT_{HLM_{1}}\left(C_{m,n}\right)$
3, 1	0.3020	0
4, 2	1.7877	1.0304
5, 3	2.8921	1.6854
6, 4	3.9665	2.5545
7, 5	5.1119	3.55542

**Table 25:** 2^*nd*^ Leap Zagreb & 2^*nd*^ Leap Hyper Zagreb Entropy values for *C*_*m*,*n*_.

[*m*, *n*]	$ENT_{LM_{2}}\left(C_{m,n}\right)$	$ENT_{HLM_{2}}\left(C_{m,n}\right)$
3, 1	0	0
4, 2	2.3567	0.8668
5, 3	3.1844	1.0219
6, 4	4.0818	1.8134
7, 5	5.6542	2.7193

**Table 26:** Second Leap Zagreb & Second Leap Hyper Zagreb Entropy values for *C*_*m*,*n*_.

[*m*, *n*]	$ENT_{LM_{1}}\left(C_{m,n}\right)$	$ENT_{LM_{2}}\left(C_{m,n}\right)$	$ENT_{LM_{2}}\left(C_{m,n}\right)$
3, 1	0.6020	0	0.1874
4, 2	1.7877	2.3567	0.9867
5, 3	2.8921	3.1844	1.6713
6, 4	3.9665	4.0818	2.5445
7, 5	5.1119	5.6542	3.5517

To compare how Entropy increases as the structure of *C*_*m*,*n*_ expands. Above graphical representation shows that Wiener index and Wiener polarity Entropy increases monotonically but Wiener entopy increases rapidly as *C*_*m*,*n*_ expands.

To compare how Entropy increases as the structure of *C*_*m*,*n*_ expands. Above graphical representation shows that forgotten index and Forgotten co-index Entropy increases monotonically but forgotten co-index entropy increases more rapidly as *C*_*m*,*n*_ expands. Here value of forgotten coindex entropy is negtive it shows that more ordered information. We often think that as value of entropy is positively increased it shows disorder behavior. But on the other hand negtinve value of entropy shows more order. We have taken the values of Forgottan index from the paper Shazia et al.

To compare how Entropy increases as the structure of *C*_*m*,*n*_ expands. Above graphical representation shows that First Leap Zagreb and First Leap Hyper Zagreb Entropy increases monotonically but in the beginning First Leap Hyper Zagreb entropy increases slightly less than First Leap Zagreb entropy. As *C*_*m*,*n*_ expands First Leap Zagreb entropy increases continuously. But behavior for Leap Hyper Zagreb entropy is much less straight forward show.

To compare how Entropy increases as the structure of *C*_*m*,*n*_ expands. Above graphical representation shows that Second Leap Zagreb and Second Leap Hyper Zagreb Entropy increases monotonically but in the beginning First Leap Zagreb entropy increases slightly more than First Leap Hyper Zagreb entropy. As *C*_*m*,*n*_ expands First Leap Zagreb entropy increases rapidly but for Leap Hyper Zagreb entropy shows some times slightly little change in the behavior.

To compare how Entropy increases as the structure of *C*_*m*,*n*_ expands. Above graphical representation shows that First Second and third Leap Zagreb Entropy increases monotonically but as *C*_*m*,*n*_ expands mostly all these entropies shows similar behavior but second leap zagreb entropy increased rapidly some time slow down then increased.

## 11. Conclusions

The information entropy of the regular dendrimer asymptotically rises with the generation number and it has been examined both theoretically and experimentally. The information entropy of common dendrimers, such as fractal and Cayley tree type dendrimers is found to rise with the number of generations. The system’s information capacity must thus be temporarily raised in order to move it from one state of informational stability to another. Entropy which describes a comparable loss of data in information transmission networks has found use in the field of information theory.

The reader is encouraged to study the generalized Wiener polarity index and some other distance-based topological descriptors such as Szeged-type indices and find their entropies for Fractal and Cayley tree type dendrimers. Many related chemical structures can be studied to compute entropy of degree and distance based topological indices, that is still open for investigation and as challenge for researchers.
