# Correction: On distance-based topological indices and co-indices of fractal-type molecular graphs and their respective graph entropies

**DOI:** 10.1371/journal.pone.0316743

**Published:** 2024-12-30

**Authors:** Mehar Ali Malik, Muhammad Imran, Muhammad Adeel

There are errors in the author affiliations. The correct affiliations are as follows:

Mehar Ali Malik^1^, Muhammad Imran^2^, Muhammad Adeel^3^

**1** Department of Basic Sciences and Humanities, College of Electrical and Mechanical Engineering, National University of Sciences and Technology (NUST), Rawalpindi, Pakistan, **2** Department of Mathematical Sciences, United Arab Emirates University, Al Ain, United Arab Emirates, **3** Department of Mathematics, Riphah International University, Lahore, Pakistan.
